# Food-Based Dietary Guidelines in Countries of the Eastern Mediterranean Region: A Comparison and an Update

**DOI:** 10.3390/ijerph22121790

**Published:** 2025-11-26

**Authors:** Ayoub Al-Jawaldeh, Mandy Taktouk, Rhea Fahd, Jennifer Ayoub, Lara Nasreddine

**Affiliations:** 1 Regional Office for the Eastern Mediterranean (EMRO), World Health Organization (WHO), Cairo 7608, Egypt; aljawaldeha@outlook.com; 2 Nutrition and Food Sciences Department, Faculty of Agricultural and Food Sciences, American University of Beirut, Beirut 11-0236, Lebanon; mt86@aub.edu.lb (M.T.); rf91@aub.edu.lb (R.F.)

**Keywords:** food-based dietary guidelines, healthy diet, health promotion, Eastern Mediterranean Region

## Abstract

Food-Based Dietary Guidelines (FBDGs) are key tools for providing comprehensive, evidence-based recommendations for healthy eating using simple and understandable holistic diet approaches. This study aims to review national FBDGs in the Eastern Mediterranean Region (EMR), examine and compare their dietary and lifestyle recommendations, and explore the inclusion of environmental sustainability considerations. The Food and Agriculture Organization’s (FAO’s) global online FBDGs repository, Google Scholar, and ministry websites were consulted. Results showed that national FBDGs were available in 12 out of the 22 EMR countries (54.5%). The main purpose of these FBDGs was the promotion of general health by emphasizing the adoption of healthy dietary and lifestyle patterns. The various FBDGs categorized their foods into five, six, or seven different groups, the main differences being related to the inclusion of legumes and nuts as a distinct food group in some guides (instead of being in the group of meats and alternative), and to the featuring of fats and oils as a separate food group. In addition to promoting healthy and varied diets, all FBDGs in the region addressed food safety (100%), physical activity (100%), meal patterns (42%), maintenance of healthy body weight (83%), sleep (50%), and emotional well-being (17%). Aspects related to environmental protection were only considered by six national FBDGs (50%), of which only four addressed sustainability (33%). The study’s findings show that a minority of the FBDGs have undertaken revisions, which are particularly important in light of the global calls for promoting diets that are healthy for both people and the planet.

## 1. Introduction

A healthy dietary pattern plays a key role in the prevention of diet-related non-communicable diseases (NCDs) and the protection against micronutrient deficiencies and their complications [1,2]. It is well established that these diet-related diseases and conditions derive, at least in part, from excessive intakes of some nutrients and inadequacies in others [1,2]. Consumers seeking evidence-based information on nutrition are often confused by the breadth of information and advice provided by different sources and sometimes even by the contradictory nature of this information [3]. Acknowledging that the promotion of healthy dietary patterns, instead of specific nutrients or foods, helps improve public understanding and adoption [4,5], the World Health Organization (WHO) and the Food and Agricultural Organization (FAO) of the United Nations advocated since 1996 for the development and implementation of food-based dietary guidelines (FBDGs) [6,7]. 

FBDGs are key tools for providing comprehensive, evidence-based recommendations for healthy eating using simple and understandable holistic diet approaches [8,9,10]. Beyond diet, FBDGs incorporate a wider scope of dimensions, including the promotion of healthy lifestyle habits and upholding the cultural appropriateness of the recommendations [10,11,12]. Since they serve as educational tools for diverse audiences, including the general public and policymakers [8,9,10], FBDGs are designed to be simple, easy to understand, and practical to implement [9,13]. Such guidelines should also be tailored to the nutritional and health status of the targeted population and ought to be updated regularly to reflect the heterogeneity in dietary patterns observed between different countries and sometimes within the same country [9,13,14]. 

Globally, more than 100 countries have developed or are currently in the process of developing FBDGs, with several having updated their FBDGs at least once [15]. Acknowledging the urgent need to shift to more sustainable diets and recognizing the policy and programmatic implications of FBDGs, the FAO has called for the development of FBDGs that promote healthy as well as environmentally sustainable diets [16]. In this context, more countries have started to incorporate sustainability considerations within their FBDGs, integrating recommendations that promote specific food practices and choices [16]. Such recommendations include an emphasis on mostly plant-based diets, seasonal and locally produced foods, reduction of food waste, and consumption of sustainably sourced fish, coupled with a reduction in the consumption of red meat, processed meat, and ultra-processed foods and beverages [16]. 

The Eastern Mediterranean Region (EMR) harbors countries with different economic and development statuses, where some countries are prospering while others are experiencing significant challenges such as political conflicts, economic disparities, and rampant food insecurity [17,18,19]. Moreover, the region grapples with the double burden of malnutrition, where overnutrition (overweight and obesity) and diet-related NCDs coexist with undernutrition and micronutrient deficiencies [17,20,21,22]. Concomitantly, countries of the region are at various stages of the nutrition transition, shifting away from traditional dietary patterns towards a more Westernized diet characterized by energy-dense foods high in saturated fats, trans fats, sugar, sodium, as well as ultra-processed foods [22,23,24]. Hence, in 2012, the WHO and FAO published general dietary guidelines for the EMR as means of providing healthy nutrition and lifestyle recommendations for the population [25]. 

Subsequently, a number of countries of the EMR have developed their own national country-specific FBDGs. Coats et al. reviewed, in 2019, five FBDGs within the Arabic-speaking region, namely the Arab Food Dome for the Arab Gulf states, the Healthy Food Palm of Saudi Arabia, the Omani Guide to Healthy Eating, the Qatar Dietary Guidelines, and the Lebanese Dietary Guidelines [26]. These Arab countries are part of the EMR, but the latter includes other Arab and non-Arab states as defined by the WHO [27]. In addition, since the review published by Coats et al. (2019), other countries in the EMR have either developed or updated their FBDGs [26]. In this context, the aim of this study is to review all the available national FBDGs from Eastern Mediterranean countries identified according to the WHO EMR Member-States classification [27]; examine and compare their dietary and lifestyle recommendations; and explore the inclusion of environmental sustainability considerations. The assessment of the methodological rigor used in developing the FBDGs, as well as the evaluation of their implementation, dissemination, and potential impact on behavioral change, are beyond the scope of this review. 

## 2. Materials and Methods

The 22 countries of the EMR were identified based on the WHO regional classification [27]. The primary source of information was the FAO’s global online FBDGs repository [28], which publishes updated FBDGs as they are developed or revised by countries and endorsed by a governmental entity. The FAO repository includes country pages, which generally present the official name of the national FBDGs, the publication year (usually the most recent revision), the intended audience or target group(s), the food guide (description of the graphic representation), and the key messages that are provided. Additional resources including Google Scholar and ministry websites were visited to identify relevant FBDGs not found on the FAO website. 

For this review, the most updated FBDG of each country was selected to be reviewed. The assessment was principally conducted on the FBDGs published in English, except for the countries whose FBDGs are only published in the local language. 

The information that was extracted from each FBDG included the following:

- FBDG title, year of publication, years since the last revision, and available languages.
- Intended purpose and intended use of FBDG (target population; whether it was indicated that it could be used by policymakers or healthcare practitioners, etc.; whether it included special population groups).
- Pictorial illustration.
- Food groups included in the guidelines and whether quantitative recommendations were provided (number of servings from various food groups and definitions of servings). For further comparative purposes, food group and quantitative recommendations from the United States Department of Agriculture (USDA) Dietary Guidelines and selected European national guidelines were also examined.
- Dietary intake/behavior recommendations.
- Recommendations related to health-related behaviors (including the maintenance of healthy body weight, engaging in physical activity, promoting adequate sleep, and emotional well-being).
- Non-dietary recommendations (including food and water safety and food labeling).
- Cost and affordability considerations.
- Environmental sustainability considerations.

## 3. Results

### 3.1. Overview of the National FBDGs in Countries of the EMR 

National FBDGs were available in 12 out of the 22 countries of the EMR (54.5%). These 12 countries include Afghanistan, Bahrain, Iran, Jordan, Kuwait, Lebanon, Oman, Pakistan, Palestine, Qatar, Saudi Arabia, and the United Arab Emirates (UAE) [29,30,31,32,33,34,35,36,37,38,39,40]. Based on the World Bank classification, one country is classified as low income (Afghanistan), five as middle-income countries (Jordan, Lebanon, Pakistan, and Palestine are lower middle-income countries while Iran is an upper middle-income country), and six as high-income countries (Bahrain, Kuwait, Oman, Qatar, Saudi Arabia, and the UAE) (Table 1) [41].

Table 2 and Table 3 present the national FBDGs of the 12 EMR countries [29,30,31,32,33,34,35,36,37,38,39,40]. The guidelines of six EMR countries (Afghanistan, Iran, Lebanon, Qatar, Saudi Arabia, and the UAE) were published within the FAO’s global online FBDGs repository [28,29,31,34,38,39,40]. The guidelines of six countries (Bahrain, Jordan, Oman, Pakistan, and Palestine) were available on the website of their respective Ministries of Health [30,32,35,36,37]. The FBDGs of Kuwait were recently published by the Public Authority of Food and Nutrition [33]. 

Across the reviewed countries, the development of FBDGs was led by national governmental entities, mainly Ministries of Health or equivalent bodies, often in collaboration with international partners such as the FAO and WHO EMRO (Supplementary Table S1) [29,31,33,34,35,36,38,39,40,42,43,44,45,46,47]. Lebanon represents an exception, where the guidelines were developed by an academic institution and later endorsed by the Ministry of Public Health. In nearly all other countries—such as Afghanistan, Bahrain, Iran, Jordan, Oman, Pakistan, Qatar, Saudi Arabia, and the UAE—the process was characterized by multisectoral engagement, bringing together government ministries, academia, professional societies, and international organizations. In line with the guidance provided by the FAO and WHO for the development of FBDGs [6,48], most countries adopted a multi-step, evidence-based methodology that included a comprehensive review of scientific evidence on food consumption patterns and public health priorities, coupled with expert consultations. 

Libya published national dietary guidelines for chronic disease management [49], and Yemen presented some dietary guidelines for Yemeni people within their National Nutrition Strategy document [50]. Therefore, these two guidelines were excluded from this review. 

Only Iran and Oman have updated their original FBDGs. Although Iran had frequent revisions of its FBDG, the latest revision was performed a decade ago (2015) [31]. The oldest (and unrevised) FBDGs were published by Saudi Arabia (2012) [39], followed by Lebanon (2013) [34], Qatar (2015) [38], and Afghanistan (2016) [29]. More recent FBDGs (published within the past five years) included the UAE (2019) [40], Jordan (2020) [32], Palestine (2021) [37], Kuwait (2023) [33], Oman (2024) [35], and Bahrain (2025) [30]. 

Of the twelve national FBDGs, four (33%) are available in English only [29,33,36,39], three (25%) in their local language only (Arabic) [32,37,40], and five (42%) in both English and the local language (Arabic, Persian) [30,31,34,35,38]. The main purpose stated by all of these FBDGs (100%) is the promotion of general health by emphasizing the adoption of healthy, culture-specific, dietary, and lifestyle patterns. Most FBDGs (75%) also included disease prevention within their purpose statements, namely non-communicable and diet-related diseases. 

In terms of the target population, four guides (Bahrain, Kuwait, Oman, and Qatar) were intended to be used by the general population [30,33,35,38], and one guide (Lebanon) specified the target population as Lebanese adults [34]. Four guides (Iran, Palestine, Saudi Arabia, and the UAE) widened their targeted age range to two years and above [31,37,39,40]. Three guides specified their target population to include infants, children, pregnant and lactating women (Afghanistan, Jordan, and Pakistan) [29,32,36], as well as adolescents and older adults (Jordan and Pakistan) [32,36]. All FBDGs also indicated their potential use by policymakers, healthcare practitioners, nutritionists, and/or nutrition educators. 

Specific recommendations to special population or vulnerable groups were often included, such as infants (Palestine and Qatar) [37,38], children and adolescents (Bahrain, Kuwait, Oman, and Saudi Arabia) [30,33,35,39], pregnant and/or lactating women (Bahrain, Kuwait, Lebanon, Oman, Palestine, Qatar, and Saudi Arabia) [30,33,34,35,37,38,39], women of reproductive age (Kuwait, Lebanon, Oman, and Palestine) [33,34,35,37], older adults (Bahrain, Kuwait, Lebanon, Oman, Palestine, Qatar, and Saudi Arabia) [30,33,34,35,37,38,39], vegetarians (Bahrain, Jordan, Kuwait, Lebanon, Oman, Palestine, and Qatar) [30,32,33,34,35,37,38], and lactose intolerant consumers (Bahrain, Jordan, Kuwait, Lebanon, Palestine, and Qatar) [30,32,33,34,37,38]. Kuwait and Oman also included recommendations for physically active individuals and those with glucose-6-phosphate dehydrogenase (G6PD) deficiency, respectively [33,35]. In addition, Lebanon’s FBDGs provided advice for postmenopausal women and individuals with a weakened immune system [34].

**Figure 1:**
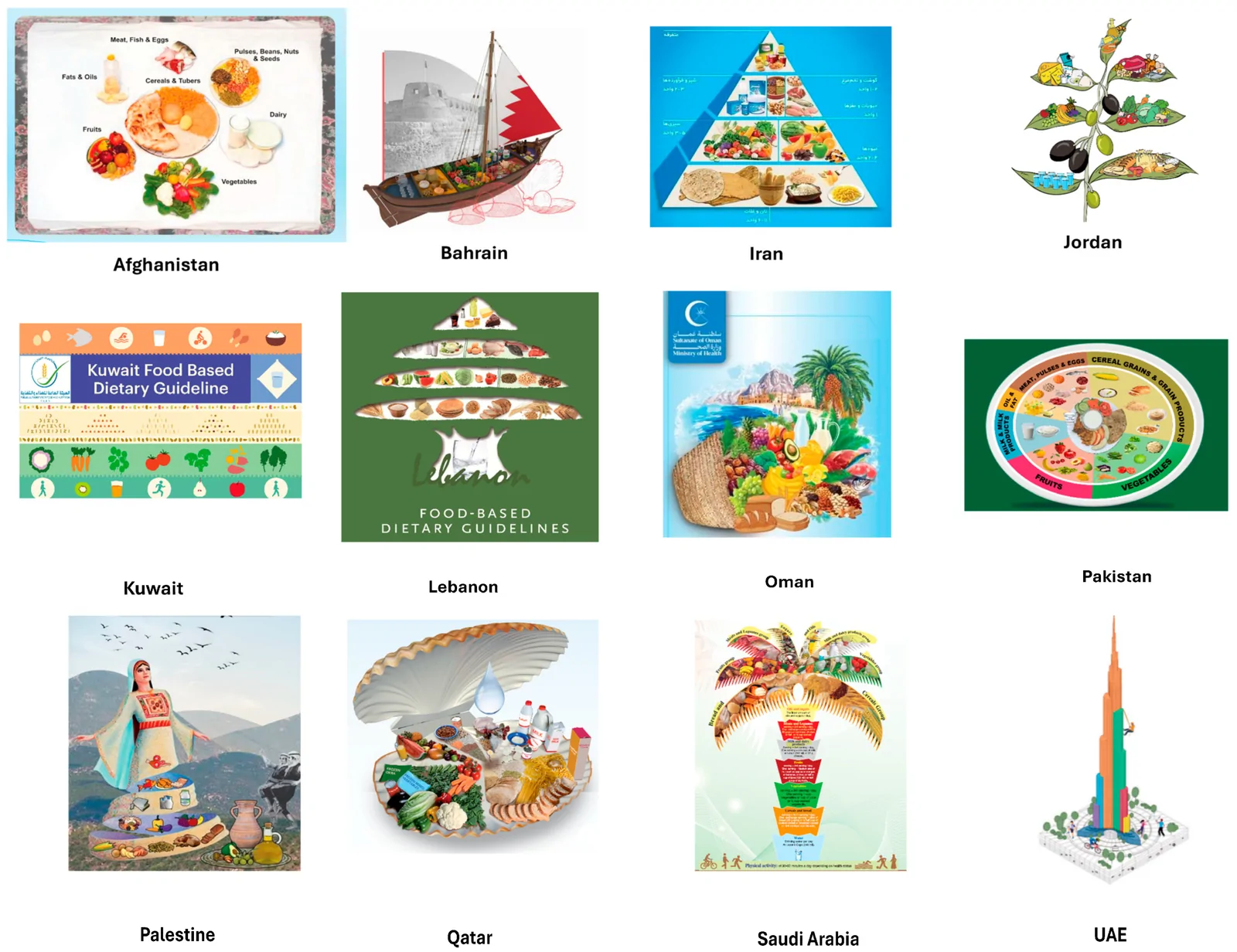
Pictorial illustrations of the national FBDGs of countries in the EMR. Abbreviations: EMR: Eastern Mediterranean Region; FBDGs: food-based dietary guidelines; UAE: United Arab Emirates. The pictorial illustrations were extracted from the national FBDGs, as shown in the following list of resources: Afghanistan [29]; Bahrain [30]; Iran [31]; Jordan [32]; Kuwait [33]; Lebanon [34]; Oman [35]; Pakistan [36]; Palestine [37]; Qatar [38]; Saudi Arabia [39]; UAE [40].

As shown in Figure 1, the pictorial illustrations used in the national FBDGs varied among the countries (Figure 1). Iran illustrated the food pyramid model [31], while Jordan (olive tree) [32], Lebanon (cedar) [34], Palestine (traditional dress “thobe”) [37], and Saudi Arabia (palm tree) [39] embedded the concept of the food pyramid within their cultural symbols/representation. Oman adopted a food basket-based illustration [35], while Pakistan adopted a plate-based illustration [36]. Qatar used the concept of the MyPlate within its cultural illustration of a seashell [38], while Kuwait showed icons pertinent to food items and physical activity [33]. The FBDGs of Afghanistan and the UAE developed other cultural representations, such as a tablecloth and Burj Khalifa, respectively [29,40]. 

### 3.2. Food Groupings

**Figure 2:**
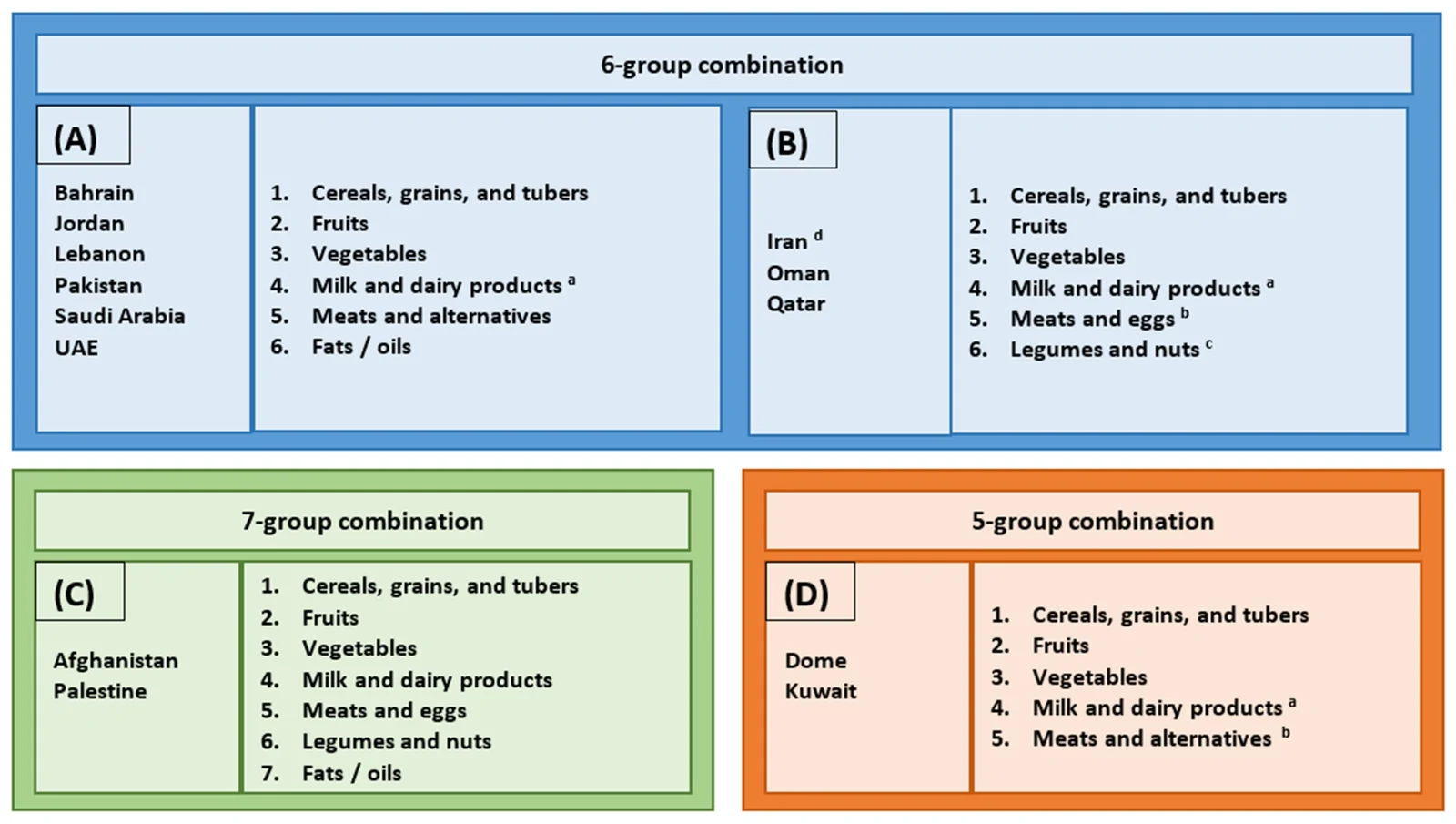
Numbers and types of food groups used in the national FBDGs of the EMR countries. Abbreviations: EMR: Eastern Mediterranean Region; FBDGs: food-based dietary guidelines. ^a^ Qatari and Kuwait FBDGs include dairy alternatives. ^b^ Qatari and Kuwaiti FBDGs include meat alternatives such as nuts and seeds. ^c^ Qatari FBDG excludes nuts. ^d^ Iran categorized oils as part of their miscellaneous food group.

The various FBDGs in the EMR categorized their foods into five, six, or seven different groups. As shown in Figure 2, the most common categorization was that of the six-group type, specifically that shown in panel (A), which included the following: 1. cereals, grains, and tubers; 2. fruits; 3. vegetables; 4. milk and dairy products; 5. meats and alternatives; and 6. fats and oils. These food groups were adopted by Bahrain, Jordan, Lebanon, Pakistan, Saudi Arabia, and the UAE [30,32,34,36,39,40], and are in line with the food groups used in the USDA dietary guidelines [51], as well as the ones included in the regional EMR FBDGs [25]. Iran, Oman, and Qatar have also adopted six food groups in their FBDGs but these groups (shown in panel B) were relatively different than the aforementioned ones [31,35,38]. More specifically, for Iran and Oman, legumes and nuts were considered as a distinct food group (instead of being included in the group of meats and alternative), while the group of fats and oils was not featured [31,35]. For Qatar, legumes were considered as a distinct food group, while nuts remained within the meat and alternatives group [38].

Legumes and nuts were also featured as a separate food group in the guides from Afghanistan and Palestine, which adopted a seven-group combination (panel C) [29,37]. As for Kuwait, it adopted a five-food-group categorization, with these groups being identical to those shown in panel A, with the exception of the fats and oils group, which was excluded [33]. These five food groups are the same as the ones used in the regional FBDGs for the Gulf Cooperation Council (GCC) countries (the Dome) [26]. In each of the combinations, common food groups included cereals, grains, and tubers; fruits; and vegetables. The milk and dairy products food group was also common but was further extended in the guides of Qatar and Kuwait to include dairy alternatives [33,38]. Saudi Arabia addressed sugar within the fats and oils food group [39]; Lebanon addressed both sugar and salt within the fats and oils category [34], while Iran combined oils in its miscellaneous category [31]. 

For further comparative purposes, the food groups adopted by selected European Union (EU) countries were reviewed, as shown in Appendix A. The EU FBDGs generally classify foods into five main groups: (1) starchy foods, (2) fruits and vegetables, (3) milk and dairy products, (4) meats and alternatives, and (5) fats and oils [52,53,54,55,56,57]. Unlike the FBDGs in the EMR and the US, the European guidelines combine fruits and vegetables into a single group.

### 3.3. Quantitative Food Groups’ Recommendations 

Five national FBDGs (42%; Afghanistan, Bahrain, Kuwait, Oman, and the UAE) presented their food groups’ recommendations quantitatively (Appendix A) [29,30,33,35,40], meaning that the guides provided clear recommendations on the daily number of servings from each food group as well as clear definitions of the serving size per food group. Another five guides (42%; Jordan, Lebanon, Pakistan, Palestine, and Saudi Arabia) presented quantitative recommendations for all food groups except for fats/oils for which the recommendations focused on limiting the intake without quantification [32,34,36,37,39]. The Qatari FBDG provided qualitative guidelines for all the food groups except for fruits and vegetables (for which quantitative recommendations were given) [38]. Iran’s FBDGs developed qualitative guidelines for each of its food groups [31]. 

### 3.4. Dietary Intake/Behavior Recommendations Tackled by the National FBDGs in the EMR

#### 3.4.1. Varied and Balanced Diet

**Figure 3:**
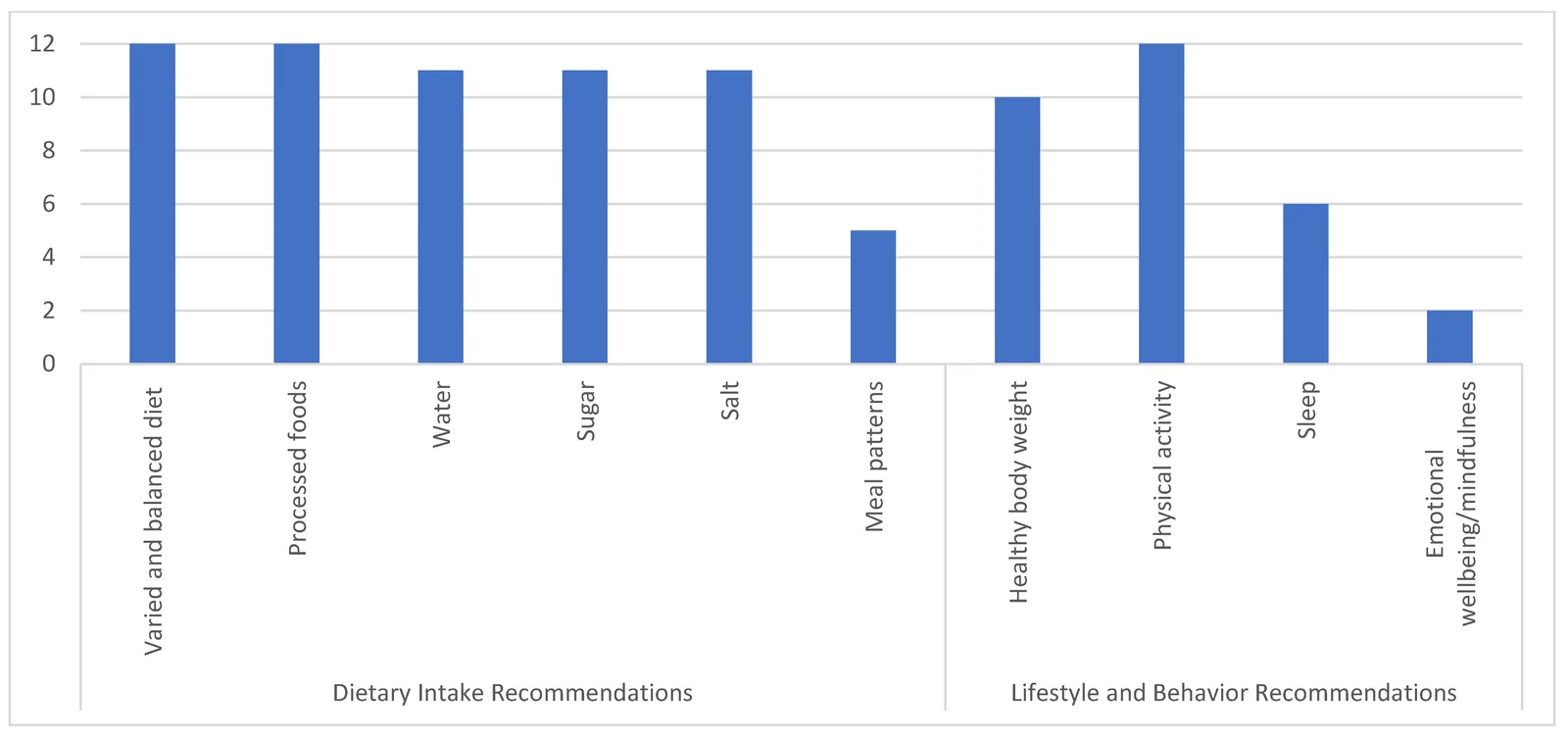
The number of FBDGs including various dietary, lifestyle, and behavior recommendations in their guidelines. Abbreviations: FBDGs: food-based dietary guidelines.

As shown in Table 4 and Figure 3, all of the reviewed FBDGs recommended that consumers adhere to a varied and balanced diet, promoting the consumption of different food groups throughout the day. Almost all guides (11) included a specific guideline devoted to this topic, whereas Pakistan’s FBDG presented this concept as a dietary message, recommending the consumption of all food groups as part of maintaining a normal body weight. Culture-specific examples of locally available and affordable foods were provided for the various food groups to foster adherence. Supplementary Table S2 provides of examples of culture-specific foods featured in the FBDGs. 

In addition to concepts pertinent to the diversification of the diet, eight countries (Afghanistan, Bahrain, Jordan, Lebanon, Oman, Pakistan, Palestine, and Saudi Arabia) dedicated a main guideline to fruits and vegetables, recommending the inclusion of a variety of fruits and vegetables within daily consumption [29,30,32,34,35,36,37,39]. As for legumes, a food group that is part of the traditional cuisine in countries of the region [58], Pakistan has dedicated one specific guideline for the promotion of legumes consumption [36], while five countries (Bahrain, Jordan, Lebanon, Oman, and Palestine) included legumes along with nuts and seeds/as part of a vegetarian alternative) [30,32,34,35,37].

#### 3.4.2. Processed Foods

Three guides (Afghanistan, Kuwait, and Pakistan) allocated specific guidelines for limiting the consumption of highly processed foods [29,33,36]. More specifically, Afghanistan and Pakistan presented this topic within the same guideline of limiting salt and fatty food intake [29,36], while Kuwait devoted a specific guideline for the promotion of more natural and minimally processed foods [33] (Table 4). 

Other FBDGs also presented recommendations throughout their guides to switch to fresh, minimally processed foods as means of decreasing the intake of fat, salt, sugar, and/or potentially carcinogenic compounds. For instance, Bahrain’s FBDG focused on replacing processed meats with vegetarian alternatives such as legumes and unsalted nuts [30]; Oman directed its guideline towards eating more fish instead of processed meats [35]; Qatar provided, within the content of decreasing salt, sugar, and fat, brief advice to avoid processed meats and to choose fresh and homemade foods [38]. Moreover, Jordan, Lebanon, and Palestine provided brief statement recommendations within their guidelines to limit the consumption of processed foods to help decrease the consumption of fats, trans fat, saturated fat, salt, and carcinogenic compounds [32,34,37]. Iran recommended reducing the intake of solid fat and fried foods [31], and Saudi Arabia and the UAE presented very brief advice on limiting processed items [39,40].

#### 3.4.3. Beverages

Eleven out of the twelve FBDGs (Afghanistan, Bahrain, Jordan, Kuwait, Lebanon, Oman, Pakistan, Palestine, Qatar, Saudi Arabia, and the UAE) featured a specific guideline dedicated to water [29,30,32,33,34,35,36,37,38,39] (Table 4 and Figure 3). Quantitative recommendations were provided by the FBDGs of Afghanistan, Kuwait, Lebanon, Qatar, Saudi Arabia, and the UAE, which were as follows: 8 cups per day for Afghanistan [29], 8–12 cups for Qatar [38], 6–8 cups for the UAE [40], and at least 6 cups of water daily for Saudi Arabia [39]. Kuwait and Lebanon specified an intake of 3.7 L and 2.7 L of water for men and women, respectively [33,34]. Bahrain, Jordan, Oman, Pakistan, and Palestine included recommendations for total fluids intakes according to age [30,32,35,36,37]. Moreover, some countries provided recommendations regarding the intake of fluids among pregnant and/or lactating women (Bahrain and Oman for pregnant women [30,35], Kuwait and Pakistan for lactating women [33,36], and Jordan and Lebanon for pregnant and lactating women [32,34]).

As for the types of beverages, Bahrain, Jordan, Oman, Pakistan, Palestine, Qatar, and the UAE recommended limiting the daily consumption of sugar-sweetened beverages (SSBs) [30,32,35,36,37,38,40]. Kuwait and Lebanon provided recommendations to replace SSBs with water and other healthy and non-caloric beverages [33,34]. Afghanistan and Qatar recommended that SSBs be avoided, while Saudi Arabia did not address SSBs [29,38]. 

#### 3.4.4. Sugar

Bahrain, Lebanon, Oman, and the UAE recommended limiting sugar intake to less than 10% of total energy intake [30,34,35,40]. Bahrain and the UAE also highlighted that decreasing sugar intake to less than 5% of total energy intake may offer additional health benefits [30,40]. Jordan, Pakistan and Palestine recommended limiting the intake of free sugars to 10% of total energy intake [32,36,37], while Kuwait recommended limiting daily sugar intake to 25 g (i.e., six teaspoons) [33]. Afghanistan, Qatar, and Saudi Arabia did not present any specific recommendations on the intake of sugar [29,38,39]. Throughout the various national FBDGs, advice was given to limit desserts, sweets (including Traditional sweets such as Knafa, Baklawa, Maamoul, Luqaimat), cakes, pastries and baked goods, pies, breakfast cereals, candies, confectionaries, sugary snacks, biscuits, puddings, chocolates, flavored yogurt, ice cream, honey, jams, jellies, some salad dressings and sauces, as well as sweetened beverages (fruit juices, syrup-based beverages, carbonated beverages, sports drinks, energy drinks, sweetened coffee and tea, flavored milk).

#### 3.4.5. Salt

As shown in Table 4 and Figure 3, five countries had a dedicated guideline to salt (Jordan, Lebanon, Oman, Pakistan, and Palestine) [32,34,35,36,37], while the other countries tackled salt as part of more comprehensive guidelines. For instance, in Afghanistan, salt was tackled within the guideline “Use less salt and eat fewer fatty foods and highly processed foods” [29], and in Qatar, salt was addressed as part of the Guideline “Limit sugar, salt, and fat” [38]. 

All countries defined a quantitative recommendation for the intake of salt. Afghanistan and Pakistan recommended an upper intake limit of 5 g of salt (2.5 g of sodium) a day [29,36]; Bahrain, Jordan, Kuwait, Oman, Palestine, and Qatar recommended an upper limit of 5 g of salt (<2 g of sodium) [30,32,33,35,37,38]; Lebanon and Saudi Arabia recommended an upper limit of 2.3 g of sodium [34,39]; and the UAE recommended an upper limit of 6 g of salt (i.e., one teaspoon; 2.3 g of sodium) [40].

Additional recommendations were presented by Bahrain and Lebanon, whereby individuals with high blood pressure or other health complications were advised to limit their intake of sodium to <1500 mg per day (equivalent to 2/3 teaspoon of salt) [30,34]. Palestine recommended similar reductions in sodium intake to <1400 mg per day (i.e., 3.5 g of salt) [37]. Kuwait also emphasized that the salt intake should be limited under the age of two years [33]. Throughout the various FBDGs, advice was given to limit high-salt foods such as processed foods (ready-to-eat meals; processed meats such as bacon, mortadella, sausages, and salami; smoked meats; processed cheeses); high-salt traditional fish; fast foods; regular, traditional breads and pastries; pickled vegetables; instant soups and noodles; high-salt condiments and sauces; salty snacks such as chips, pretzels, crackers, salted nuts, and seeds; popcorn; canned foods; olives; and salted yogurt-based beverages.

#### 3.4.6. Meal Patterns

Five national FBDGs (42%) provided advice on the importance of healthy and regular meal patterns. Jordan, Lebanon, Bahrain, and Qatar included this recommendation as part of their guidelines on healthy body weight [30,32,34,38]. Specifically, Jordan and Lebanon geared this recommendation to underweight individuals seeking to gain weight [32,34], whereas Bahrain and Qatar geared it to overweight children and adolescents as a strategy to reach a healthier body weight [30,38]. Qatar also included brief advice on healthy meal patterns within its guideline on limiting nutrients of concern [38]. The importance of regular meal patterns was also highlighted within the guideline pertinent to taking care of the family in Qatar’s and Bahrain’s FBDGs [30,38], whereby further recommendations on the importance of family meals were also provided. Oman’s FBDGs promoted regular meal patterns with its guideline on varied and balanced diets [35], and Lebanon provided brief advice on healthy snacking [34]. Pakistan included brief recommendation on the significance of breakfast consumption for the pediatric population [36]. 

### 3.5. Lifestyle and Behavior Recommendations Tackled by the National FBDGs in the EMR

Table 5 provides a summary of the various dietary behaviors and lifestyle recommendations addressed by the FBDGs in the EMR. A brief description is included below: 

#### 3.5.1. Healthy Body Weight

Ten national FBDGs (83%) (Bahrain, Iran, Jordan, Kuwait, Lebanon, Oman, Pakistan, Palestine, Qatar, and Saudi Arabia) included a specific guideline devoted to the maintenance of a healthy body weight, while also tackling this topic within other recommendations throughout the guides [30,31,32,33,34,35,36,37,38,39] (Table 5 and Figure 3).

#### 3.5.2. Physical Activity

All of the reviewed FBDGs included a guideline on physical activity (Table 4 and Table 5), presenting the benefits of regular physical activity and practical tips on how to be more active. When describing the different types of physical activity types and intensities, most FBDGs provided examples for each exercise category. All FBDGs (100%) provided specific recommendations (quantitative) for the adult population. Recommendations for other population groups varied across FBDGs. For children and youth, all FBDGs except those of Lebanon, Palestine, and Saudi Arabia presented quantitative recommendations for this age group. For pregnant women, Bahrain provided quantitative recommendations, and Jordan, Lebanon, and Oman presented general qualitative advice [30,32,34,35]. For the older adults population, Bahrain, Oman, and Pakistan provided quantitative recommendations [30,35,36], while Jordan, Kuwait, Lebanon, and Qatar presented general qualitative advice [32,33,34,38]. For adults whose target is weight loss rather than weight maintenance, Jordan, Lebanon, Oman, and Palestine presented specific quantitative recommendations [32,34,35,37]. 

#### 3.5.3. Sleep

Six national FBDGs (50%) (Bahrain, Jordan, Kuwait, Palestine, Qatar, and the UAE) have, therefore, discussed the importance of adequate sleep as part of a heathy lifestyle [30,32,33,37,38,40]. Jordan and Palestine developed a specific guideline recommending enough sleep and rest on a daily basis [32,37]. These two FBDGs, along with Kuwait and the UAE, provide information on the adequate number of sleep hours per age group [33,40]. Bahrain, Qatar, and Kuwait recommended healthy sleeping habits as essential components of weight management and/or correlated obesity with sleep complications [30,33,38]. Kuwait considered adequate sleep as an essential factor for adopting as a healthy lifestyle [33].

#### 3.5.4. Emotional Well-Being/Mindfulness

The aspect of emotional well-being and mindfulness was discussed in two FBDGs (17%) (Bahrain and Kuwait) [30,33]. Bahrain developed a specific guideline on eating homemade foods with others to promote enjoyment and connection with cultural heritage, and tips on how to practice mindful eating were presented [30]. Kuwait recommended avoiding distractions during mealtimes and focusing on being more mindful while eating as crucial components of shaping a healthy lifestyle [33].

### 3.6. Non-Dietary Recommendations Presented in the FBDGs of the EMR Countries 

#### 3.6.1. Safe Food and Water

All FBDGs included advice on food safety handling practices, and methods, with more focused recommendations presented in the FBDGs of Bahrain, Jordan, Kuwait, Lebanon, Oman, Palestine, Qatar, Saudi Arabia, and the UAE [30,32,33,34,35,37,38,39,40]. Moreover, advice on the use and consumption of clean water was included in the FBDGs of Afghanistan, Jordan, Kuwait, Lebanon, Oman, and Pakistan [29,32,33,34,35,36] (Table 6).

#### 3.6.2. Cost and Affordability 

Inherently, all the FBDGs are based on the inclusion of locally available and affordable foods, as highlighted in the preamble of most of the reviewed FBDGs. Some additional concepts of affordability were further tackled in three (25%) national FBDGs (Bahrain, Lebanon, and Pakistan) [30,34,36]. Pakistan estimated the costs of their suggested sample menus and provided general advice on healthy, economical meals [36]. Lebanon provided brief advice on dietary supplements as an alternative for individuals who cannot afford fish [34]. Bahrain introduced the aspect of saving money within its tenth guideline and associated it with decreased food waste [30]. As for Qatar, its FBDGs indicated that choosing water over other beverages when dining out helps save costs [38].

#### 3.6.3. Food Labeling

The importance of food labels in selecting healthier choices was stated in ten national FBDGs (83%) [29,30,32,33,34,35,36,37,38,40]. The extent to which this aspect was discussed varied among guides, from whole guidelines to brief statements. More specifically, Jordan, Palestine, and Kuwait developed an entire guideline on food labels, elaborating how to read and interpret them to make healthier purchases and to understand claims pertinent to specific nutrients of concern such as sodium, fat, and/or sugar content [32,33,37]. Afghanistan, Oman, and Qatar also generously discussed food labels within one of their guidelines and provided instructions related to reading and analyzing labels of foods and beverages to make healthier choices [29,35,38]. In other guides, the topic of food labels was briefly mentioned. In the FBDGs of Lebanon and the UAE, reading about claims pertinent to specific nutrients of concern such as fat, sodium, and sugar content was presented [34,40]. The Bahraini guide provided brief advice on the importance of reading labels [30], and Pakistan presented general definitions without elaborating instructions [36]. 

#### 3.6.4. Environmental Factors

Aspects related to environmental protection (including sustainability and food waste) were only considered by six national FBDGs (50%), at varying degrees [30,32,33,35,36,38]. Bahrain, Kuwait, and Qatar developed a whole guideline for the protection of the environment while emphasizing sustainable healthy diets and food waste reduction [30,33,38]. Bahrain and Qatar provided general environmentally friendly tips such as eating more plant-based food products, considering nutritious lentils as plant-based proteins, opting for unprocessed foods such as whole grains while reducing processed foods, considering local food products, and reducing waste [30,33]. Kuwait, however, focused solely on reducing waste, while providing guidance on recycling and avoiding single-use plastics [33]. Pakistan presented a general concept of the need for sustainable food production systems and decreased food loss and food waste [36]. The need for environmental protection was also briefly discussed in Jordan (the impact of plastic bottles on the environment) [32] and Kuwait (reducing food waste and recycling food packaging) [33].

## 4. Discussion

Governments in the EMR are grappling with the social and economic impacts of the increasing burden of NCDs, particularly obesity and its associated conditions, coupled with the persistence of micronutrient deficiencies and hidden hunger [17,20,21,22]. Hence, research and public health policies aimed at promoting better nutrition and healthier lifestyles are essential to prevent nutritional excesses or deficits and combat physical inactivity in the population [59,60,61]. FBDGs are amongst the strategies that can effectively reach the public, when their content and messages are clear, easy to understand, and culturally compatible with local dietary habits and lifestyle [6,62]. Importantly, as originally outlined in the 1998 FAO/WHO guidance document [6] and more recently reiterated by the FAO [62], FBDGs should tackle the pre-identified country-specific diet-related problems, as well as the food consumption patterns that may be contributing to these problems. This paper provides an overview of all the existent FBDGs in the EMR, a region that harbors a double burden of malnutrition while encompassing countries with divergent economic and food security statuses.

The paper identified 12 countries (out of 22) (54.5%) with FBDGs. This percentage is lower than that reported from other regions such as Europe (64%) [5]. All countries of the GCC (Bahrain, Kuwait, Oman, Qatar, Saudi Arabia, and the UAE), which are classified as high-income countries by the World Bank, have developed national FBDGs. In contrast, amongst the five low-income countries in the region, only Afghanistan has developed FBDGs, and amongst the eight lower middle-income countries, only 50% (four countries) have developed FBDGs. These findings are in line with those reported by previous studies on the implementation of health policies in relation to countries’ economic profiles [63]. For instance, Mackenbach and Mckee, 2013 [64] showed that, in European countries, national income was amongst the significant predictors of national performance in several areas of nutrition policy, such as fruit and vegetable consumption and alcohol-related policies. Indeed, it is expected that richer countries would have more resources to develop and implement a higher number of health-related policies as compared to poorer ones. 

A total of 5/12 (42%) of the identified FBDGs based their graphical illustrations on the pyramid or a culture-specific derivative of the pyramid. This is lower than the proportion of countries adopting the pyramid illustration in Europe (67%) [5]. In most of these pyramid-based illustrations, foods that should represent the largest proportions of a recommended healthy diet are shown at the base of the pyramid, whereas foods to be consumed more sparingly are featured at the top [5]. Some other FBDGs adopted a circular pictorial representation of their food guide, hence being rather based on the MyPlate illustration. In addition to food groups, Jordan, Kuwait, Lebanon, Oman, Qatar, and Saudi Arabia showed water in their pictorial illustrations, while only Kuwait, Saudi Arabia, and the UAE showed physical activity-related icons in their illustrations. 

There were considerable similarities in the food groupings adopted by the various national FBDGs, which rather reflects the cultural homogeneity between countries of the region. The main observed differences were related to the inclusion of legumes and nuts as a distinct food group in some of the guides (instead of being included in the group of meats and alternative), and to the featuring of fats and oils as a separate food group. The food groups adopted by several countries in the region (Bahrain, Jordan, Lebanon, Pakistan, Saudi Arabia, and the UAE) [30,32,34,36,39,40], were aligned with those used in the USDA Dietary Guidelines [51]. A key difference between the FBDGs of the EMR and those of European countries relates to the classification of fruits and vegetables, as European guidelines typically combine them into a single food group [52,53,54,55,56,57].

All of the reviewed FBDGs emphasized the consumption of a varied and balanced diet, promoting the consumption of different food groups throughout the day. Acknowledging that the consumption of diversified dietary patterns plays a key role in the prevention of micronutrient deficiencies, this guideline is of direct relevance to countries of the region and the persistent burden of micronutrient deficiencies amongst its population. Recent reviews showed that deficiencies in folate, iron, and vitamin D remain prevalent among children, adolescents, women of childbearing age, pregnant women, and the elderly in many EMR countries, including those in advanced nutrition transition stages [21,65]. Another review focusing on children showed that dietary intake studies have documented inadequate intakes of several micronutrients including iron, calcium, zinc, folic acid, vitamin A, and vitamin D in this age group [65]. In addition, varied and balanced diets can contribute to the prevention of obesity and related NCDs, which is also of direct pertinence to the disease burden in the EMR [66,67]. In fact, the prevalence estimates of NCDs in the region are increasing at an alarming rate and exceeding at times those reported from developed countries [66,67,68,69]. Overall, it is estimated that NCDs account for over 50% of annual deaths (2.2 million deaths) and 60% of the disease burden in the EMR [70]. 

The high NCD burden may be directly linked to the escalating prevalence of overweightness and obesity in the region [17,18,20,22]. The region’s weighted prevalence of adult overweightness and obesity were estimated at 27% and 24%, respectively, and that amongst school-age children at 16.5% and 4.8%, respectively [22]. On the other hand, low-income countries and those facing conflict or political instability grapple with a high burden of undernutrition, including underweightness [22]. Many countries also harbor a double burden of over- and under-nutrition in different segments of the population [22], highlighting the need to tackle this public health concern. Accordingly, ten national FBDGs (83%) included a specific guideline devoted to the maintenance of a healthy body weight.

Acknowledging the increasingly documented link among highly processed foods, obesity, and suboptimal nutritional status [71,72], almost all the FBDGs discussed the importance of limiting the consumption of such foods. Reading and understanding food labels was also tackled by ten FBDGs in an effort to equip consumers with the knowledge needed to choose healthier options when choosing to consume packaged/processed foods. In addition, five FBDGs (42%) provided advice on the importance of maintaining healthy meal patterns. In fact, research has shown that regular meal patterns are associated with improved nutrient intakes and diet quality, as well as healthier weight status in all age groups [73,74,75], while also supporting cognitive function [76] and overall well-being [77,78].

In addition to unhealthy dietary patterns, physical inactivity is another key risk factor for obesity and NCDs. A recent systematic review and meta-analysis of the prevalence of physical activity in the Middle East and North Africa region, which includes many of the EMR countries, reported that 49.2% of adults and 74.4% of children and adolescents were not sufficiently active [79]. Acknowledging the importance of physical activity for overall health and well-being, all of the reviewed FBDGs included a specific guideline devoted to physical activity. Other lifestyle factors that were also addressed included sleep, which was tackled by six national FBDGs (50%). The inclusion of sleep in the guidelines reflects the increasing amount of evidence linking insufficient sleep to a number of neural and hormonal changes that play a role in the development of obesity and several NCDs ultimately impacting morbidity and mortality [80,81]. Sleep also plays a key role in improving mental health [82], and this is of crucial relevance to the EMR, where depression reached one of the highest prevalence rates globally (4·3%) [83]. In this context, two FBDGs also tackled additional dimensions linked to emotional well-being, family bonds, and mindfulness within their recommendations. 

It is important to note that in the past, the development of FBDGs was dominated by dietary and disease prevention recommendations. However, in an era where climate change and environmental degradation pose the biggest threat to the population, health and environmental dimensions cannot be considered separately anymore [84]. According to the FAO, considering the policy and programmatic implications of FBDGs, the development and incorporation of recommendations that promote specific food practices and choices represent a clear opportunity for tackling sustainability, mainly in its nutritional and environmental dimensions [84]. In this context, Oman, Bahrain, and Qatar highlighted dietary patterns and advice that are generally of low impact on the environment, while fostering good health. Jordan, Kuwait, and Pakistan also included some sustainability considerations. The promotion of the consumption of legumes, a food group that is part of the traditional diet in countries of the region [58], was highlighted by several of the national FBFGs as a protein-rich food and an alternative to animal-based protein foods. Countries that have not integrated environmental sustainability within their guidelines are urged to revise (or develop) their FBDGs while aligning health promotion aspects with sustainability considerations. Examples of such recommendations include the promotion of a mostly plant-based diet, prioritizing seasonal and local foods, minimizing food waste, choosing fish from sustainable sources, and reducing the intake of red and processed meats, ultra-processed foods, and sugary drinks [84].

This study showed that 12 countries in the EMR developed FBDGs; however, it did not examine an equally important aspect—i.e., whether these guidelines were effectively disseminated and implemented. In this context, the FAO recommends that the implementation of FBDGs should involve all relevant stakeholders—including government, civil society, and the private sector—and be supported by coordinated communication, capacity building, and multisectoral plans to ensure their broad understanding, acceptance, and integration into national policies [85]. Future studies should, therefore, investigate how EMR countries planned and operationalized the implementation and dissemination of their FBDGs. 

In addition, evaluating the impact of FBDGs on population dietary behaviors is crucial, as emphasized by the FAO and WHO, which recommend that countries monitor and assess the effects of FBDGs on dietary behaviors, food systems, and health outcomes to determine whether the recommendations are achieving their intended impact [6]. Global evidence suggests that greater adherence to dietary guidelines may be associated with healthier cardiometabolic profiles and improved nutrient adequacy [86,87]. Available evidence from the region remains limited. In Qatar, a study assessing adherence to the national FBDGs and its association with cardiometabolic risk factors among adults found that adherence was inversely associated with elevated waist circumference (OR = 0.88; 95% CI: 0.82–0.95) and metabolic syndrome (OR = 0.84; 95% CI: 0.74–0.96) [88]. However, the Qatari study identified major gaps between national FBDG recommendations and prevailing consumption patterns, revealing that over 83% of adults did not meet recommendations for vegetables, fruits, whole grains, legumes, and fiber intake, while 47% reported regular fast-food consumption [88]. In Saudi Arabia, Halawani et al. (2019) found that average adherence to the national dietary guidelines among adults was only 26%, with the majority reporting intakes below recommended levels for vegetables (98%), dairy (91%), fruits (88%), grains (88%), water (78%), protein (62%), and sugar (54%) [89]. Similarly, in Pakistan, overall adherence to dietary guidelines was poor, with no participant achieving a full adherence score (5/5) and only 1% scoring 4/5, with adherence to the fruit intake recommendations being the lowest [90]. 

Thus, although this review did not assess implementation or impact, the available evidence underscores the need for countries in the region to strengthen efforts toward more effective implementation, dissemination, and evaluation of their FBDGs. In this context, Saudi Arabia has developed and published a framework for the evaluation of its national FBDGs [42], while for other countries evidence pertinent to evaluation plans is less clear. It is important to acknowledge that, for several lower-income countries in the EMR, the development, implementation, evaluation, and revision of FBDGs (and nutrition policies more broadly) are especially challenging due to ongoing conflict, political instability, disrupted food systems, funding deficits, and institutional capacity constraints [91,92].

This study has several limitations. The analyses in this review may be incomplete for countries whose FBDGs were developed in foreign, non-Arabic languages and not published in English (for example, Iran, where the guidelines are available only in Persian). As this review did not aim to evaluate the scientific rigor of the methodologies adopted in the development of FBDGs, we did not assess the quality and thoroughness of the processes followed by the various countries—such as whether they were based on systematic reviews or utilized a Grading of Recommendations Assessment, Development, and Evaluation (GRADE) approach. Nonetheless, since these FBDGs have already been officially released by the respective countries, their inclusion in this review is essential for assessing the content and scope of topics addressed by FBDGs in the EMR. 

## 5. Conclusions

This study showed that 12 out of 22 EMR countries (54.5%) have developed national FBDGs, with only 2 countries (Iran and Oman) having revised/updated their originally developed guides. The study also provided a comparative analysis of the graphical illustrations, the food groups adopted by each country, their quantitative intake recommendations, the dietary and lifestyle behaviors addressed in the FBDGs, and additional aspects such as food and water safety, food labeling, and environmental considerations. Notably, only about one third of the reviewed FBDGs incorporated an environmental sustainability perspective. This underscores the need for most EMR countries to revise their FBDGs, particularly in light of global calls to promote diets that are healthy for both people and the planet. In addition, updates and adaptations may be needed in countries that have recently experienced conflict, political instability, or rising food insecurity. Countries that have not yet developed dietary guidelines are also encouraged to formulate FBDGs tailored to their cultural contexts, with the aim of strengthening nutrition education and improving the populations’ dietary practices. In developing or revising FBDGs, particular attention should be given to establishing clear implementation and evaluation plans and frameworks, which should also be examined in future studies focusing on the region.

## Figures and Tables

**Table 1:** Classification of the 22 EMR countries by income and the presence of national FBDGs.

Country	Income Classification	Presence of National FBDGs
Afghanistan	Low-income	Yes
Bahrain	High-income	Yes
Djibouti	Lower middle-income	No
Egypt	Lower middle-income	No
Iran	Upper middle-income	Yes
Iraq	Upper middle-income	No
Jordan	Lower middle-income	Yes
Kuwait	High-income	Yes
Lebanon	Lower middle-income	Yes
Libya	Upper middle-income	No
Morocco	Lower middle-income	No
Oman	High-income	Yes
Pakistan	Lower middle-income	Yes
Palestine	Lower middle-income	Yes
Qatar	High-income	Yes
Saudi Arabia	High-income	Yes
Somalia	Low-income	No
Sudan	Low-income	No
Syrian Arab Republic	Low-income	No
Tunisia	Lower middle-income	No
UAE	High-income	Yes
Yemen	Low-income	No

Abbreviations: FBDGs: food-based dietary guidelines; EMR: Eastern Mediterranean Region; UAE: United Arab Emirates.

**Table 2:** Overview of national FBDGs in countries of the Eastern Mediterranean Region.

	Afghanistan [29]	Bahrain [30]	Iran [31]	Jordan [32]	Kuwait [33]	Lebanon [34]	Oman [35]	Pakistan [36]	Palestine [37]	Qatar [38]	Saudi Arabia [39]	UAE [40]
**FBDG title ^a^**	National food-based dietary guidelines for Afghans: A manual	The food national-based dietary guidelines for the Kingdom of Bahrain (Arabic: الإرشادات الغذائية البحرينية)	Food-based dietary guidelines for Iran (Persian: ایران رهنمودهای غذایی).	Food-based dietary guideline for Jordanians (Arabic: الدليل الارشادي الغذائي للأردنيين).	Kuwait food-based dietary guidelines (KFBDG)	The food-based dietary guideline manual for promoting healthy eating in the Lebanese adult population (Arabic: دليل الإرشادات التوجيهية الغذائية لتشجيع الغذاء السليم لدى البالغين اللبنانيين)	The Omani guide to healthy eating (Arabic: الدليل العماني للغذاء الصحي)	Pakistan dietary guidelines for better nutrition	Palestinian food-based dietary guidelines	Qatar dietary guidelines (Arabic: الدلائل الإرشادية للتغذية لدولة قطر).	Dietary guidelines for Saudis: The healthy food palm (Arabic: النخلة الغذائية الصحية)	United Arab Emirates Dietary guidelines (Arabic: الدليل الارشادي الوطني للتغذية
**Year of publication ^b^**	2016	2023	2015	2020	2023	2013	2023	2019	2021	2015	2012	2019
If not the first version, year of previous publications	-	-	2015 2006 1996 1993 1990	-	-	-	2009	-	-	-	-	-
**Years since last revision**	Nine years	-	Ten years	Five years	-	Twelve years	-	Six years	Four years	Ten years	Thirteen years	Six years
**Available language(s)**	English	English and Arabic	Persian and English	Arabic	English	English and Arabic	English and Arabic	English	Arabic	English and Arabic	English	Arabic
**FBDG purpose**												
*Healthy diet*	√	√	√	√	√	√	√	√	√	√	√	√
*Disease prevention*	-	√	-	-	√	√	√	√	√	√	√	√
**Pictorial illustration**	Tablecloth	Not available	Pyramid	Olive tree (pyramid)	Food items and physical activity icons	Cedar (pyramid)	Food basket	Plate	Thobe (pyramid)	Seashell (plate)	Palm tree (pyramid)	Burj Khalifa

Abbreviations: FBDG: food-based dietary guideline; UAE: United Arab Emirates. ^a^ When available, the official name of the FBDG was extracted from the FAO’s global online FBDGs repository [28]. For non-English FBDG titles, the title in the local language was either provided by the authors (Bahrain, Jordan, Lebanon, and Oman), extracted from the FAO’s global online FBDGs repository (Iran and Saudi Arabia), or translated by the authors of this review who speak the Arabic language (Palestine). ^b^ Or year of first FBDG if no previous versions were published. √: indicates the presence of the mentioned criteria; -: indicates the absence of the mentioned criteria.

**Table 3:** Target population and intended use of the FBDGs in countries of the Eastern Mediterranean Region.

	Afghanistan [29]	Bahrain [30]	Iran [31]	Jordan [32]	Kuwait [33]	Lebanon [34]	Oman [35]	Pakistan [36]	Palestine [37]	Qatar [38]	Saudi Arabia [39]	UAE [40]
**Target population**	General population, which also includes infants and children; PW and LW	General population	General population over two years of age	General population, which also includes infants and children; Adolescents; PW and LW; Older adults	General population	Adult population	General population	General population, which also includes infants and children; Adolescents; PW and LW; Older adults	General population two years and above	General population	General population two years and above	General population two years and above
**To be used by policymakers and/or HCPs and/or nutritionists and/or nutrition educators**	√	√	√	√	√	√	√	√	√	√	√	√
**Special population groups addressed**	-	Children and adolescents PW Older adults Vegetarians Lactose intolerant	-	Vegetarians Lactose intolerant	Children and adolescents WRA, PW, and LW Older adults Vegetarians Lactose intolerant Physically active individuals	WRA, PW, LW, and postmenopausal women Older adults Vegetarians Lactose intolerant Individuals with weakened immune systems	Children and adolescents WRA, PW, and LW Older adults Vegetarians Individuals with G6PD deficiency	-	WRA, PW, and LW Older adults Vegetarians Lactose intolerant Infants	LW Older adults Vegetarians Lactose-free consumers Infants	Children and adolescents PW and LW Older adults	-

Abbreviations: FBDG: food-based dietary guideline; G6PD: glucose-6-phosphate dehydrogenase; HCPs: healthcare practitioners; LW: lactating women; PW: pregnant women; UAE: United Arab Emirates; WRA: women of reproductive age. √: indicates the presence of the mentioned criteria; -: indicates the absence of the mentioned criteria.

**Table 4:** Table of contents for the national FBDGs identified in EMR countries, with the specific guidelines.

Afghanistan [29]	Guideline 1. Eat different types of food daily Guideline 2. Eat different types of fruit and vegetables daily Guideline 3. Eat lean meat, poultry, fish, eggs, and dairy products Guideline 4. Reduce sugar intake and avoid sweet carbonated beverages Guideline 5. Use less salt, and eat fewer fatty foods and highly processed foods Guideline 6. Pregnant and lactating women should increase intake of all the food groups daily, especially foods that are rich in iron Guideline 7. Give infants only breast milk for the first six months of life Guideline 8. From six months onward, feed infants and young children different nutritious foods in addition to breast milk and continue breastfeeding until 24 months Guideline 9. Use clean and safe water for handwashing, drinking, and food preparation Guideline 10. If you live a sedentary life, do some physical activity for at least 20–30 min daily
Bahrain [30]	Guideline 1. Look after your body weight today to enhance your health tomorrow Guideline 2. Move more. Exercise not only optimizes your body, it enhances your mind and mood Guideline 3. Try varying and balancing your diet. This a lifestyle, not “all or nothing” Guideline 4. Keep it simple by sticking to more fruits and vegetables Guideline 5. Switch to healthier animal proteins, while emphasizing low-fat milk and dairy and fish/sea food Guideline 6. Choose vegetarian alternatives to red and processed meat such as legumes and unsalted nuts Guideline 7. Pay attention to your intake of salt and sugar, especially the hidden ones Guideline 8. Stay hydrated with water and healthy fluids Guideline 9. Observe the recommendations for safe food production and consumption Guideline 10. Contribute to protecting the environment, feeding the hungry, and saving money by decreasing food waste Guideline 11. Eat homemade foods with others to promote enjoyment and connect with cultural heritage
Jordan [32]	Guideline 1. Maintain a healthy body weight Guideline 2. Be physically active every day Guideline 3. Drink enough safe water every day and make it your first choice Guideline 4. Follow a healthy and nutritious dietary pattern with the five food groups Guideline 5. Eat adequate amounts of seasonal vegetables and fruits daily Guideline 6. Eat different kinds of whole grains Guideline 7. Consume legumes daily and enjoy some unsalted nuts and seeds Guideline 8. Consume sufficient amounts of milk and dairy products daily Guideline 9. Consume lean red meats, fish, and poultry Guideline 10. Avoid consuming fats in large quantities Guideline 11. Limit the intake of foods and beverages high in sugar, especially added sugar Guideline 12. Limit the intake of table salt and high-salt foods Guideline 13. Read the nutrition labels when purchasing food products Guideline 14. Follow food safety guidelines when preparing, consuming, and storing food Guideline 15. Ensure daily adequate sleep and rest and maintain a healthy lifestyle Guideline 16. Obtain nutritional information from specialized sources
Kuwait [33]	Guideline 1. Eat a healthy and balanced diet consisting of the five food groups every day Guideline 2. Make natural and minimally processed foods the base of your daily meals Guideline 3. Drink adequate amount of water every day Guideline 4. Limit your intake of fat, added sugar, and salt Guideline 5. Know your food: Read the food labels to make healthier choices Guideline 6. Practice safe and clean food handling methods Guideline 7. Adopt a healthy lifestyle and protect your environment Guideline 8. Maintain a healthy body weight Guideline 9. Move more and stay active
Lebanon [34]	Guideline 1. Enjoy and maintain a healthy body weight Guideline 2. Be physically active every day Guideline 3. Eat a variety of nutritious foods every day for a balanced diet Guideline 4. Eat cereals, especially whole grains, as a basis of daily meals Guideline 5. Enjoy more fruit and vegetables daily Guideline 6. Consume legume-based dishes regularly and enjoy some unsalted nuts and seeds Guideline 7. Consume low-fat milk and dairy products every day Guideline 8. Consume at least two servings of fish, including fatty fish, every week Guideline 9. Consume lean red meat and poultry Guideline 10. Limit intake of sugar, especially added sugar from sweetened foods and beverages Guideline 11. Limit intake of solid fats and replace with vegetable oils Guideline 12. Limit intake of table salt and high-salt foods Guideline 13. Drink plenty of safe water every day Guideline 14. Eat safe food
Oman [35]	Guideline 1. Maintain a healthy body weight for better health and well-being Guideline 2. Be active and exercise Guideline 3. Vary your diet: variety is the spice of life Guideline 4. Know your portion size Guideline 5. Eat more vegetables and fruits every day Guideline 6. Choose whole grains Guideline 7. Eat legumes, and add unsalted nuts and seeds to your diet Guideline 8. Eat more fish, less red meat, and avoid processed meat Guideline 9. Choose low-fat, unsweetened dairy products Guideline 10. Switch to healthier fats and oils Guideline 11. Choose food with less salt Guideline 12. Hold back on sugar Guideline 13. Drink plenty of safe water and eat safe food
Pakistan [36]	Guideline 1. Maintain normal body weight by consuming all food groups and performing regular physical activity Guideline 2. Half of your daily cereals intake should include whole grains Guideline 3. Eat five servings of fresh vegetables and fruits a day Guideline 4. Take two to three servings of milk and milk products in a day Guideline 5. Consume meat and meat products, fish, and eggs in moderation Guideline 6. Encourage consumption of pulses to attain healthy growth Guideline 7. Consume fortified flour, grains, and their products Guideline 8. Limit consumption of edible oil and fat in cooking Guideline 9. Reduce sugar intake, and limit intake of soft drinks, confectionaries, bakery products, and commercial fruit drinks Guideline 10. Limit salt in cooking and always use iodized salt Guideline 11. Limit consumption of fatty foods and highly processed foods Guideline 12. Change sedentary lifestyle to physically active lifestyle Guideline 13. Exclusively breastfeed the baby in the first six months and continue breastfeeding along with complementary feeding at least for two years Guideline 14. Women should increase intake of all the food groups daily, especially foods that are rich in iron, and take extra care during pregnancy and lactation Guideline 15. Drink plenty of water each day Guideline 16. Read nutrition labeling on packaged food products
Palestine [37]	Guideline 1. Maintain an ideal body weight Guideline 2. Drink sufficient quantities of clean water Guideline 3. Follow a healthy and balanced dietary pattern that includes all food groups Guideline 4. Eat adequate amounts of vegetables and fruits daily Guideline 5. Eat all kinds of whole grains Guideline 6. Consume legumes daily and enjoy some unsalted nuts and seeds in moderation Guideline 7. Consume sufficient amounts of milk and dairy products daily Guideline 8. Consume a variety of lean red meats, fish, and poultry Guideline 9. Avoid consuming fats and oils in large quantities Guideline 10. Limit the intake of foods and beverages high in sugar, especially added sugar Guideline 11. Limit the intake of table salt and high-salt foods Guideline 12. Read the nutrition labels when purchasing food products Guideline 13. General food safety guidelines when preparing, consuming, and storing food Guideline 14. Ensure daily adequate sleep and rest Guideline 15. Obtain food and nutritional information from specialized sources
Qatar [38]	Guideline 1. Eat a variety of healthy choices from the 6 food groups Guideline 2. Maintain a healthy weight Guideline 3. Limit sugar, salt, and fat Guideline 4. Be physically active Guideline 5. Drink plenty of water Guideline 6. Adopt safe and clean food preparation methods Guideline 7. Eat healthy while protecting the environment Guideline 8. Take care of your family
Saudi Arabia [39]	Guideline 1. Enjoy a variety of food items from major food groups daily Guideline 2. Choose whole grains Guideline 3. Consume a variety of fruits and vegetables Guideline 4. Limit the intake of foods with a high content of saturated fatty acids, cholesterol, salt, and sugar Guideline 5. Achieve and maintain a healthy body weight Guideline 6. Drink water Guideline 7. Purchase, prepare, cook, and store food in ways to ensure food safety Guideline 8. Be physically active
UAE [40]	Guideline 1. Support and emphasize a healthy lifestyle through a healthy diet and physical activity for all Guideline 2. Maintain the consumption of healthy foods throughout the different stages of life Guideline 3. Reduce calories by lowering sugar and fat, especially saturated and trans fats, and lowering salt intake Guideline 4. Consume a variety of nutritious and energy dense foods and beverages Guideline 5. To adopt healthy dietary patterns, replace unhealthy foods with healthy foods Guideline 6. Food safety

Abbreviations: EMR: Eastern Mediterranean Region; FBDGs: food-based dietary guidelines; UAE: United Arab Emirates.

**Table 5:** Lifestyle and behavior recommendations presented in the FBDGs of the EMR countries.

	Afghanistan [29]	Bahrain [30]	Iran [31]	Jordan [32]	Kuwait [33]	Lebanon [34]	Oman [35]	Pakistan [36]	Palestine [37]	Qatar [38]	Saudi Arabia [39]	UAE [40]
Healthy body weight	-	Look after your body weight today to enhance your health tomorrow.	Eat in amounts so that your ideal body weight will be maintained.	Maintain your healthy weight.	Maintain a healthy body weight.	Enjoy and maintain a healthy body weight.	Maintain a healthy body weight for better health and well-being.	Maintain normal body weight by consuming all food groups and performing regular physical activity.	Maintain an ideal weight.	Maintain a healthy weight.	Achieve and maintain a healthy body weight.	-
Varied and balanced diet	Eat different types of foods daily.	Try varying and balancing your diet. This a lifestyle, not “all or nothing”.	Select a balanced, varied diet.	Follow a healthy and varied diet from the five food groups.	Eat a healthy and balanced diet consisting of the five food groups every day.	Eat a variety of nutritious foods every day for a balanced diet.	Vary your diet: variety is the spice of life.	Maintain normal body weight by consuming all food groups and performing regular physical activity.	Follow a healthy and balanced diet that includes all food groups.	Eat a variety of healthy choices from the six food groups.	Enjoy a variety of food items from major food groups daily.	Diversify the intake of nutrient-dense and high-density foods and beverage.
The importance of meal patterns	-	Special population children and adolescents: Help your children develop healthy eating behaviors and dietary patterns. Eating in isolation is linked to many unhealthy dietary patterns such as stress eating and binge eating.	-	For underweight: Eat multiple and separate meals so that there are three snacks in addition to three main meals.	-	For underweight: Eat small and frequent meals throughout the day.	Maintain a healthy eating pattern, with three main meals (and two snacks).	-	-	Build and model healthy patterns for your family. Adopting healthy eating and activity patterns that can be maintained over time is a more effective way to lose weight than dieting.	-	-
Physical activity	If you live a sedentary life, do some physical activity for at least 20–30 min daily. Adults: At least 30 min of moderate to vigorous activity most days of the week.	Move more. Exercise not only optimizes your body, it enhances your mind and mood. Adults: At least 150 to 300 min of moderate-intensity aerobic physical activity OR At least 75 to 150 min of vigorous-intensity aerobic physical activity OR Equivalent combination of moderate- and vigorous-intensity activity.	Try to exercise regularly, at least three days per week, 30–40 min each time.	Be physically active daily. Adults: Engage in moderate-intensity physical activity for at least 30 min a day.	Move more and stay active. Adults: At least 150 min of moderate-intensity physical activity throughout the week or at least 75 min of vigorous-intensity physical activity throughout the week, or an equivalent combination of moderate- and vigorous-intensity activity.	Be physically active every day. Adults: At least 30 min of moderate-intensity physical activity five days per week.	Be active and exercise. Adults: At least 30 min of daily moderate-intensity physical activity five days per week. or 20 min of vigorous-intensity aerobic activity three days per week.	Change sedentary lifestyle to physically active lifestyle. Adults: At least 30 min or more moderate exercise.	Be physically active daily. Adults: At least 30 min daily, five days per week.	Be physically active. Adults: Moderate intensity at least five days per week (for at least 30 min) and/or vigorous-intensity aerobic at least three days per week (for at least 20 min).	Be physically active. Adults: Intermittent walking for 15 to 30 min, 3–4 times/week; and subsequently increase in physical activity for 30–60 min on most if not all days of the week.	Supporting and promoting a healthy lifestyle through healthy food and physical activity for all. Adults: At least 150 min of moderate-intensity aerobic physical activity OR At least 75 min of vigorous-intensity aerobic physical activity OR Equivalent combination of moderate- and vigorous-intensity activity.
Sleep	-	Tips for achieving and maintaining a healthy body weight among children and adolescents; ensuring adequate sleep,	-	Get enough sleep and rest daily and maintain a healthy lifestyle.	Adopt a healthy lifestyle and protect your environment: Healthy sleeping habits	-	-	-	Get enough sleep and rest daily.	For weight loss: Getting enough sleep is essential. Recent research suggests that a lack of sleep is related to overweight and obesity. Getting enough sleep may also help you to have enough energy to exercise.	-	Adequate number of sleep hours per age group.
Emotional well-being/mindfulness	-	Eat homemade foods with others to promote enjoyment and connect with cultural heritage.	-	-	Avoid distractions during mealtimes and focus on being more mindful while eating.	-	-	-	-	-	-	-

Abbreviations: EMR: Eastern Mediterranean Region; FBDGs: food-based dietary guidelines; UAE: United Arab Emirates; -: indicates the absence of the mentioned criteria.

**Table 6:** Non-dietary recommendations presented in the FBDGs of the EMR countries.

	Afghanistan [29]	Bahrain [30]	Iran [31]	Jordan [32]	Kuwait [33]	Lebanon [34]	Oman [35]	Pakistan [36]	Palestine [37]	Qatar [38]	Saudi Arabia [39]	UAE [40]
**Food and water safety**												
Food safety handling practices	√	√	√	√	√	√	√	√	√	√	√	√
Clean water use and consumption	√	-	-	√	√	√	√	√	-	-	-	-
**Environmental factors**	-	√	-	√	√	-	√	√	-	√	-	-
Sustainability	-	√	-	-	-	-	√	√	-	√	-	-
Food waste	-	√	-	-	√	-	√	√	-	√	-	-
**Cost and affordability**	-	√	-	-	-	√	-	√	-	-	-	-
**Food labeling**	√	√	-	√	√	√	√	√	√	√	-	√

Abbreviations: EMR: Eastern Mediterranean Region; FBDGs: food-based dietary guidelines; UAE: United Arab Emirates; √: indicates the presence of the mentioned criteria; -: indicates the absence of the mentioned criteria.

## Data Availability

The original contributions presented in this study are included in the article.

## Supplementary Materials

The following supporting information can be downloaded at: https://www.mdpi.com/article/10.3390/ijerph22121790/s1, Table S1: The process of FBDG development in countries of the Eastern Mediterranean Region; Table S2. Examples of culture-specific foods in the FBDGs.

## Correction Statement

This article has been republished with a minor correction to the copyright information. This change does not affect the scientific content of the article.

## Abbreviations

The following abbreviations are used in this manuscript:

EMR	Eastern Mediterranean Region
EU	European Union
FAO	Food and Agricultural Organization
FBDGs	food-based dietary guidelines
G6PD	glucose-6-phosphate dehydrogenase
GCC	Gulf Cooperation Council
GRADE	Grading of Recommendations Assessment, Development, and Evaluation
HCP	healthcare practitioners
LW	lactating women
NCDs	non-communicable diseases
PW	pregnant women
SSBs	sugar-sweetened beverages
UAE	United Arab Emirates
USDA	United States Department of Agriculture
WHO	World Health Organization
WRA	women of reproductive age

## Appendix A

**Table A1:** Food groups’ daily recommendations (servings per food group per day) for countries with the 7-group categorization.

Seven-Group Categorization		
	Afghanistan [29] Three Energy Levels (1300, 2200, 2800 kcals)	Palestine [37] (Serving Recommendations Vary per Age and Gender)
Cereals, grains, and tubers	2.5, 6, 8 servings	3–8 servings
Fruits	2.5, 2.5, 3 servings	1–2 servings
Vegetables	2.5, 3, 3 servings	1–3 servings
Milk and dairy products	2, 3.5, 4 servings	2–3 servings
Meats and eggs	1.5, 2, 2.5 servings	2–6 servings
Legumes and nuts	0.5, 1.5, 2 servings	2–6.5 servings
Fats/Oils	4.5, 5.5, 7 servings	Limited intake

**Table A2:** Food groups’ daily recommendations (servings per food group per day) for countries with the 6-group categorization.

**Six-group categorization (A)**								
	**USDA 2020–2025 [52]** **(Based on a 2000 kcal Regular Diet) ***	**WHO-EMR** **[25]**	**Bahrain** **[30]**	**Jordan** **[32]**	**Lebanon** **[34]**	**Pakistan** **[36]**	**Saudi Arabia** **[39]**	**UAE [40]** **(Serving Recommendations Vary per Age and Gender)**
Cereals, grains, and tubers	6 oz equivalents	180 g equivalent	6 servings	At least 6 servings	At least 6 servings, with at least ½ being whole grain	4–5 servings	6–11 servings	3–8 oz
Fruits	2 cup equivalents	4 servings	2 servings	2 servings	2 servings	2–3 servings	2–4 servings	1–2 cups
Vegetables	2.5 cup equivalents	5 servings	2.5 servings	2–3 servings	2–3 servings	2–3 servings	3–5 servings	1–3 cups
Milk and dairy products	3 cup equivalents	3 cup equivalents	3 servings	3 servings	3 servings	2–3 servings	2–4 servings	2–3 cups
Meat and alternatives	5.5 oz equivalents	160 g equivalent	5.5 servings	5–6.5 servings	5–6.5 servings	2–3 servings	2–3 servings	2–6.5 oz
Fats/Oils	27 g	24 g oils, 267 kcal solid fat and added sugars	27 g oils	Limited consumption	Limited consumption	Limited consumption	Lower amount possible	3–7 tsp
**Six-group categorization (B)**								
	**Iran [31]**		**Oman [35]**			**Qatar [38]**		
Cereals, grains, and tubers	Choose whole grains		6 servings			Choose whole grains		
Fruits	Eat 3 times a day		2 servings			2–4 servings		
Vegetables	Eat raw and cooked vegetables every day at main meals and snacks		2–5 servings			3–5 servings		
Milk and dairy products	Consume dairy products daily		3 servings			Maintain a daily consumption of skimmed or low-fat milk and dairy products		
Meats and eggs	Include meats, preferably chicken and fish (with skin removed), as well as eggs, in your diet		5.5 servings			Choose skinless poultry and lean cuts of meat, eat a variety of fish at least twice per week		
Legumes and nuts	Eat legumes and dishes made with legumes once a day		5.5 servings of protein per day			Eat legumes daily; choose legumes, nuts, and seeds as alternative protein sources		

* Additional serving recommendations based on different ages and energy requirements are presented in the guide.

**Table A3:** Food groups’ daily recommendations (servings per food group per day) for countries with the 5-group categorization.

	Musaiger Gulf Countries (Dome) [26]	Kuwait [33]
Cereals, grains, and tubers	6–11 servings	7 servings for women, 8 servings for men
Fruits	2–4 servings	4 servings
Vegetables	3–5 servings	5 servings
Milk and dairy products	2–3 servings	2–3 servings
Meats and alternatives	2–4 servings	2 servings for women, 3 servings for men

Abbreviations: EMR: Eastern Mediterranean Region; UAE: United Arab Emirates; USDA: United States Department of Agriculture; WHO: World Health Organization.

**Table A4:** Food groups’ recommendations for selected European countries.

	Denmark–Northern Europe [52,53]	Germany–Western Europe [53,54]	Latvia–Eastern Europe [53,55]	Spain–Southern/Mediterranean Europe [53,56]	Ireland–British Isles [53,57]
Starchy foods	At least 90 g of whole grains a day	5 servings a day, with at least 1/3 being whole grains	4–6 servings a day, particularly whole grains	3–6 servings a day	3–5 servings a day
Fruits and vegetables	6 servings (or 600 g, half of which at least are vegetables) a day	At least 5 servings a day	At least 5 servings (or 500g) a day	At least 5 servings a day	5–7 servings a day
Milk and dairy products	250–350 mL milk or dairy products and 20 g of cheese a day	2 servings a day	2–3 servings a day	Maximum 3 servings a day	3 servings a day
Meats and alternatives	- About 350 g of meat per week - 350 g of fish per week - 3 eggs per week - 30 g of nuts a day - 1–2 tablespoons of seeds a day - 100 g of legumes a day	- Up to 2 servings of meat per week but no more than 300 g of meat per week - Fish once or twice per week - 1 egg per week - 1 serving of nuts a day - At least 1 serving of legumes per week	- 2–3 servings of lean meats, fish, eggs, nuts, seeds, and legumes a day - Up to 500 g of meat per week - Fish at least twice per week	- Maximum of 3 servings of meat per week - At least 3 servings of fish per week - Up to 4 medium eggs per week - At least 3 servings of nuts per week - At least 4 servings of legumes per week	- 2 servings of meat, fish, poultry, eggs, beans, and nuts a day - Fish up to twice per week - No more than 7 eggs per week - 40 g of unsalted nuts or seeds
Fats/Oils	-	1 tablespoon of vegetable oils and 1 tablespoon of butter or margarine a day	An average of 67–80 g (i.e., no more than 25–30% of the daily energy needs)	-	-

## References

[1] Neuhouser M.L. (2019). The importance of healthy dietary patterns in chronic disease prevention. Nutr. Res..

[2] Ley S.H., Hamdy O., Mohan V., Hu F.B. (2014). Prevention and management of type 2 diabetes: Dietary components and nutritional strategies. Lancet.

[3] Liu A.G., Ford N.A., Hu F.B., Zelman K.M., Mozaffarian D., Kris-Etherton P.M. (2017). A healthy approach to dietary fats: Understanding the science and taking action to reduce consumer confusion. Nutr. J..

[4] Freeland-Graves J.H., Nitzke S. (2013). Position of the academy of nutrition and dietetics: Total diet approach to healthy eating. J. Acad. Nutr. Diet..

[5] Montagnese C., Santarpia L., Buonifacio M., Nardelli A., Caldara A.R., Silvestri E., Contaldo F., Pasanisi F. (2015). European food-based dietary guidelines: A comparison and update. Nutrition.

[6] Food and Agriculture Organization, World Health Organization (1998). Preparation and Use of Food-Based Dietary Guidelines/Report of a Joint FAO/WHO Consultation.

[7] Food and Agriculture Organization of the United Nations, World Health Organization Regional Office for the Eastern Mediterranean (2006). FAO/WHO Technical Consultation on National Food-based Dietary Guidelines.

[8] Bechthold A., Boeing H., Tetens I., Schwingshackl L., Nöthlings U. (2018). Perspective: Food-based dietary guidelines in Europe—Scientific concepts, current status, and perspectives. Adv. Nutr..

[9] Carrillo-Álvarez E., Boeckx H., Penne T., Palma Linares I., Storms B., Goedemé T. (2020). A comparison of European countries FBDG in the light of their contribution to tackle diet-related health inequalities. Eur. J. Public Health.

[10] Herforth A., Arimond M., Álvarez-Sánchez C., Coates J., Christianson K., Muehlhoff E. (2019). A global review of food-based dietary guidelines. Adv. Nutr..

[11] Springmann M., Spajic L., Clark M.A., Poore J., Herforth A., Webb P., Rayner M., Scarborough P. (2020). The healthiness and sustainability of national and global food based dietary guidelines: Modelling study. BMJ.

[12] Food and Agriculture Organization, World Health Organization Sustainable Healthy Diets—Guiding Principles. https://www.fao.org/3/ca6640en/ca6640en.pdf.

[13] Faber M., de Villiers A. (2021). Field-testing of food-based dietary guidelines. S. Afr. J. Clin. Nutr..

[14] Comerford K.B., Miller G.D., Boileau A.C., Masiello Schuette S.N., Giddens J.C., Brown K.A. (2021). Global review of dairy recommendations in food-based dietary guidelines. Front. Nutr..

[15] Food and Agriculture Organization of the United Nations Dietary Guidelines: Regions. https://www.fao.org/nutrition/education/food-dietary-guidelines/regions/en/.

[16] Fischer C.G., Garnett T. (2016). Plates, Pyramids and Planets. Developments in National Healthy and Sustainable Dietary Guidelines: A State of Play Assessment.

[17] WHO Regional Office for the Eastern Mediterranean Poor Nutrition Is Increasing Rates of Diabetes, Cancers, Heart Attacks and Stroke. WHO Launches Regional Nutrition Strategy to Help Countries Beat NCDs. https://www.emro.who.int/media/news/poor-nutrition-is-increasing-rates-of-diabetes-cancers-heart-attacks-and-stroke.html.

[18] Leppäniemi H., Ibrahim E.T., Abbass M.M., Borghi E., Flores-Urrutia M.C., Dominguez Muriel E., Gatica-Dominguez G., Kumapley R., Hammerich A., Al-Jawaldeh A. (2023). Nutrition Profile for Countries of the Eastern Mediterranean Region with different income levels: An Analytical Review. Children.

[19] Al Sharjabi S.J., Al Jawaldeh A., Hassan O.E.H., Dureab F. (2024). Understanding the Food and Nutrition Insecurity Drivers in Some Emergency-Affected Countries in the Eastern Mediterranean Region from 2020 to 2024. Nutrients.

[20] World Health Organization (2017). The Double Burden of Malnutrition: Policy Brief.

[21] Hwalla N., Al Dhaheri A.S., Radwan H., Alfawaz H.A., Fouda M.A., Al-Daghri N.M., Zaghloul S., Blumberg J.B. (2017). The prevalence of micronutrient deficiencies and inadequacies in the Middle East and approaches to interventions. Nutrients.

[22] Nasreddine L., Ayoub J.J., Al Jawaldeh A. (2018). Review of the nutrition situation in the Eastern Mediterranean Region. East. Mediterr. Health J..

[23] Naja F., Itani L., Hamade R., Chamieh M.C., Hwalla N. (2019). Mediterranean diet and its environmental footprints amid nutrition transition: The case of Lebanon. Sustainability.

[24] Aboul Enein B.H., Bernstein J., Neary A. (2016). Dietary transition and obesity in selected Arabic-speaking countries: A review of the current evidence. EMHJ-East. Mediterr. Health J..

[25] Houalla N., Al-Jawaldeh A.E., Bagchi K., Hachem F., El Ati J., Omidvar N., Cheikh L., WHO Regional Office for the Eastern Mediterranean (2012). Promoting a Healthy Diet for the WHO Eastern Mediterranean Region: User-Friendly Guide.

[26] Coats L., Bernstein J., Dodge E., Bechard L., Aboul-Enein B.H. (2019). Food-based dietary guidelines of Arabic-speaking countries: A culturally congruent profile. Public Health Nutr..

[27] WHO Regional Office for the Eastern Mediterranean Countries. https://www.emro.who.int/countries.html.

[28] Food and Agriculture Organization Food-Based Dietary Guidelines. https://www.fao.org/nutrition/education/food-dietary-guidelines/home.

[29] Food and Agriculture Organization, Ministry of Public Health Afghanistan, Ministry of Agriculture Afghanistan, Ministry of Education Afghanistan National Food-Based Dietary Guidelines for Afghans. https://www.fao.org/3/i5283e/i5283e.pdf.

[30] Ministry of Health-Bahrain Food Based Dietary Guidelines for the Kingdom of Bahrain. https://www.moh.gov.bh/Content/Upload/File/638422157715028884-225MOH---The-Food-National-Based-Dietary-Guidelines_.pdf.

[31] Ministry of Health and Medical Education Food-Based Dietary Guidelines—Iran. https://www.fao.org/nutrition/education/food-dietary-guidelines/regions/countries/iran/en/.

[32] Ministry of Health (MOH) Jordan Food Based Dietary Guideline for Jordanians (Arabic). https://www.moh.gov.jo/ebv4.0/root_storage/ar/eb_list_page/%D8%A7%D9%84%D8%AF%D9%84%D9%8A%D9%84_%D8%A7%D9%84%D8%BA%D8%B0%D8%A7%D8%A6%D9%8A_%D9%84%D9%84%D8%A7%D8%B1%D8%AF%D9%86%D9%8A%D9%86.pdf.

[33] Public Authority for Food and Nutrition Kuwait Food Based Dietary Guideline. https://www.pafn.gov.kw/assets/images/Uploads/KuwaitFoodbasedDietaryGuidlineEnglish.pdf.

[34] The American University of Beirut, Lebanese National Council for Scientific Research, Ministry of Public Health The Food-Based Dietary Guideline Manual for Promoting Healthy Eating in the Lebanese Adult Population. https://www.fao.org/nutrition/education/food-dietary-guidelines/regions/countries/lebanon/en/.

[35] Ministry of Health-Sultanate of Oman The Omani Guide for Healthy Eating. https://moh.gov.om/media/c3vhtd2y/oman-diatry-guideline-english-feb-19.pdf.

[36] Food and Agriculture Organization of the United Nations, Ministry of Planning/Development and Reform/Government of Pakistan Pakistan Dietary Guidelines for Better Nutrition. https://www.pc.gov.pk/uploads/report/Pakistan_Dietary_Nutrition_2019.pdf.

[37] Ministry of Public Health Palestine, World Health Organization (2021). Palestinian Food Based Dietary Guidelines (Arabic).

[38] The Supreme Council of Health Qatar Dietary Guidelines. https://www.fao.org/3/az908e/az908e.pdf.

[39] Ministry of Health Kingdom of Saudi Arabia Dietary Guidelines for Saudis: The Healthy Food Palm. https://www.fao.org/nutrition/education/food-dietary-guidelines/regions/countries/saudi-arabia/en/.

[40] United Arab Emirates Ministry of Health and Prevention United Arab Emirates Dietary Guidelines (Arabic). https://www.fao.org/nutrition/education/food-dietary-guidelines/regions/countries/united-arab-emirates/en/.

[41] The World Bank The World by Income and Region. https://datatopics.worldbank.org/world-development-indicators/the-world-by-income-and-region.html.

[42] Alhumaidan O.A., Alkhunein S.M., Alakeel S.A., Fallata G.A., Aldhwayan M.M., Alfaifi A.Y., Albalwi W.M., AlZeer H. (2025). Saudi healthy plate-2024: Framework for developing, modeling, and evaluating Saudi Arabia’s dietary guidelines. BMC Nutr..

[43] Government of Afghanistan Afghanistan Launches National Food Based Dietary Guidelines (FBDGs) for Improved Nutrition. https://reliefweb.int/report/afghanistan/afghanistan-launches-national-food-based-dietary-guidelines-fbdgs-improved.

[44] Naja F., Khaleel S., Alhajeri M.E., Ajlan B.Y., Abulfateh N.M., Alawadhi A.G., Bowah M.H.J., Al-Jawaldeh A. (2023). The Bahraini food based dietary guidelines: A holistic perspective to health and wellbeing. Front. Public Health.

[45] Safavi S., Omidvar N., Djazayery A., Minaie M., Hooshiarrad A., Sheikoleslam R. (2007). Development of food-based dietary guidelines for Iran: A preliminary report. Ann. Nutr. Metab..

[46] Al-Jawaldeh A. Development of Food Based Dietary Guidelines. https://www.researchgate.net/publication/339322960_DEVELOPMENT_OF_FOOD_BASED_DIETARY_GUIDLINES?channel=doi&linkId=5e4b367ba6fdccd965aee258&showFulltext=true.

[47] Musaiger A.O., Takruri H.R., Hassan A.S., Abu-Tarboush H. (2012). Food-based dietary guidelines for the Arab Gulf countries. J. Nutr. Metab..

[48] Food and Agriculture Organization of the United Nations Dietary Guidelines: Development Process. https://www.fao.org/nutrition/education/dietary-guidelines/background/development-process/en/.

[49] Al Shhawi A., World Health Organization, Ministry of Health (MOH) Libya, National Centre for Diease Control Libya (2021). Dietary Guidelines for Chronic Diseases in Libya (Arabic).

[50] Republic of Yemen/Ministry of Public Health and Population National Nutrition Strategy for Yemen (Draft). https://cmamforum.org/Pool/Resources/YEMEN-National-Nutrition-Strategy-2009.pdf.

[51] US Department of Agriculture and US Department of Health and Human Services Dietary Guidelines for Americans, 2020–2025, 9th ed. https://www.dietaryguidelines.gov/sites/default/files/2020-12/Dietary_Guidelines_for_Americans_2020-2025.pdf.

[52] Danish Veterinary and Food Administration, Ministry of Food, Agriculture and Fisheries of Denmark The Official Dietary Guidelines—Good for Health and Climate. https://www.fao.org/nutrition/education/food-dietary-guidelines/regions/countries/denmark/en/.

[53] European Commission Food-Based Dietary Guidelines in Europe: Source Documents. https://knowledge4policy.ec.europa.eu/health-promotion-knowledge-gateway/food-based-dietary-guidelines-europe-source-documents-food_en.

[54] German Nutrition Society Eat and Drink Well—Recommendations of the German Nutrition Society (DGE). https://www.fao.org/nutrition/education/food-dietary-guidelines/regions/countries/germany/en/.

[55] Ministry of Health-Latvia and Center for Disease Prevention and Control Dietary Guidelines for Adults. https://www.fao.org/nutrition/education/food-dietary-guidelines/regions/countries/latvia/en/.

[56] Spanish Agency for Food Safety and Nutrition (AESAN) of the Spanish Ministry of Consumer Affairs Healthy and Sustainable Dietary Recommendations Complemented with Physical Activity Recommendations for the Spanish Population. https://www.fao.org/nutrition/education/food-dietary-guidelines/regions/countries/spain/en/.

[57] Department of Health-Ireland Healthy Food for Life—The Healthy Eating Guidelines. https://www.fao.org/nutrition/education/food-dietary-guidelines/regions/countries/ireland/en/.

[58] Naureen Z., Bonetti G., Medori M.C., Aquilanti B., Velluti V., Matera G., Iaconelli A., Bertelli M. (2022). Foods of the Mediterranean diet: Garlic and Mediterranean legumes. J. Prev. Med. Hyg..

[59] World Health Organization, Food and Agriculture Organization (2003). Expert Report: Diet, Nutrition and Prevention of Chronic Diseases. Report of a Joint WHO/FAO Expert Consultation.

[60] Joint Programme Initiative A Healthy Diet for a Healthy life. The Vision for 2030. https://era.gv.at/public/documents/1320/Vision_paper.pdf.

[61] World Health Organization (2000). Obesity: Preventing and Managing the Global Epidemic. Report of a WHO Consultation.

[62] Food and Agriculture Organization of the United Nations Dietary Guidelines. https://www.fao.org/nutrition/education/food-dietary-guidelines/en/.

[63] Halleröd B., Rothstein B., Daoud A., Nandy S. (2013). Bad governance and poor children: A comparative analysis of government efficiency and severe child deprivation in 68 low-and middle-income countries. World Dev..

[64] Mackenbach J.P., McKee M. (2013). A comparative analysis of health policy performance in 43 European countries. Eur. J. Public Health.

[65] Nasreddine L.M., Kassis A.N., Ayoub J.J., Naja F.A., Hwalla N.C. (2018). Nutritional status and dietary intakes of children amid the nutrition transition: The case of the Eastern Mediterranean Region. Nutr. Res..

[66] Hawkes C. (2006). Uneven dietary development: Linking the policies and processes of globalization with the nutrition transition, obesity and diet-related chronic diseases. Glob. Health.

[67] Mehio Sibai A., Nasreddine L., Mokdad A.H., Adra N., Tabet M., Hwalla N. (2010). Nutrition transition and cardiovascular disease risk factors in Middle East and North Africa countries: Reviewing the evidence. Ann. Nutr. Metab..

[68] Nasreddine L., Naja F., Sibai A., Helou K., Adra N., Hwalla N. (2013). Trends in nutritional intakes and nutrition-related cardiovascular disease risk factors in Lebanon: The need for immediate action. J. Med. Liban..

[69] World Health Organization Regional Office for the Eastern Mediterranean Burden of Noncommunicable Diseases in the Eastern Mediterranean Region. https://www.emro.who.int/noncommunicable-diseases/publications/burden-of-noncommunicable-diseases-in-the-eastern-mediterranean-region.html.

[70] World Health Organization (2008). The Global Burden of Disease 2004 Update.

[71] Monda A., de Stefano M.I., Villano I., Allocca S., Casillo M., Messina A., Monda V., Moscatelli F., Dipace A., Limone P. (2024). Ultra-Processed Food Intake and Increased Risk of Obesity: A Narrative Review. Foods.

[72] García-Blanco L., de la O V., Santiago S., Pouso A., Martínez-González M.Á., Martín-Calvo N. (2023). High consumption of ultra-processed foods is associated with increased risk of micronutrient inadequacy in children: The SENDO project. Eur. J. Pediatr..

[73] Leech R.M., Worsley A., Timperio A., McNaughton S.A. (2015). Understanding meal patterns: Definitions, methodology and impact on nutrient intake and diet quality. Nutr. Res. Rev..

[74] Saltaouras G., Kyrkili A., Bathrellou E., Georgoulis M., Yannakoulia M., Bountziouka V., Smrke U., Dimitrakopoulos G., Kontogianni M.D. (2024). Associations between Meal Patterns and Risk of Overweight/Obesity in Children and Adolescents in Western Countries: A Systematic Review of Longitudinal Studies and Randomised Controlled Trials. Children.

[75] Ali M.A., Macdonald I.A., Taylor M.A. (2024). A systematic review of associations between day-to-day variability in meal pattern and body weight, components of the metabolic syndrome and cognitive function. J. Hum. Nutr. Diet..

[76] British Nutrition Foundation Food and Your Brain. https://www.nutrition.org.uk/nutrition-for/food-and-the-brain/.

[77] Mou Y., Blok E., Barroso M., Jansen P.W., White T., Voortman T. (2023). Dietary patterns, brain morphology and cognitive performance in children: Results from a prospective population-based study. Eur. J. Epidemiol..

[78] Ojo Y.A. (2016). Nutrition and Cognition in School-Aged Children: A Brief Review. Int. J. Educ. Benchmark.

[79] Chaabane S., Chaabna K., Abraham A., Mamtani R., Cheema S. (2020). Physical activity and sedentary behaviour in the Middle East and North Africa: An overview of systematic reviews and meta-analysis. Sci. Rep..

[80] Gomes S., Ramalhete C., Ferreira I., Bicho M., Valente A. (2023). Sleep patterns, eating behavior and the risk of noncommunicable diseases. Nutrients.

[81] Grandner M.A., Hale L., Moore M., Patel N.P. (2010). Mortality associated with short sleep duration: The evidence, the possible mechanisms, and the future. Sleep Med. Rev..

[82] Scott A.J., Webb T.L., Martyn-St James M., Rowse G., Weich S. (2021). Improving sleep quality leads to better mental health: A meta-analysis of randomised controlled trials. Sleep Med. Rev..

[83] GBD Mental Disorders Collaborators (2022). Global, regional, and national burden of 12 mental disorders in 204 countries and territories, 1990–2019: A systematic analysis for the Global Burden of Disease Study 2019. Lancet Psychiatry.

[84] Food and Agriculture Organization of the United Nations Dietary Guidelines and Sustainability. https://www.fao.org/nutrition/education/dietary-guidelines/background/sustainable-dietary-guidelines/en/.

[85] Food and Agriculture Organization of the United Nations Dietary Guidelines: Implementation. https://www.fao.org/nutrition/education/dietary-guidelines/background/implementation/en/.

[86] Buckland G., Taylor C.M., Emmett P.M., Northstone K. (2023). Prospective association between adherence to UK dietary guidelines in school-age children and cardiometabolic risk markers in adolescence/early adulthood in the Avon Longitudinal Study of Parents and Children (ALSPAC) cohort. Br. J. Nutr..

[87] Chaltiel D., Adjibade M., Deschamps V., Touvier M., Hercberg S., Julia C., Kesse-Guyot E. (2019). Programme National Nutrition Santé–guidelines score 2 (PNNS-GS2): Development and validation of a diet quality score reflecting the 2017 French dietary guidelines. Br. J. Nutr..

[88] Al Thani M., Al Thani A.A., Al-Chetachi W., Al Malki B., Khalifa S.A., Bakri A.H., Hwalla N., Naja F., Nasreddine L. (2018). Adherence to the Qatar dietary guidelines: A cross-sectional study of the gaps, determinants and association with cardiometabolic risk amongst adults. BMC Public Health.

[89] Halawani R., Jaceldo-Siegl K., Heskey C., Bahjri K. (2019). Saudi Population’s Adherence to the Healthy Food Palm: A Cross-sectional Study. FASEB J..

[90] Rafique I., Muhammad A.N.S., Murad N., Munir M.K., Khan A., Irshad R., Rahat T., Naz S. (2020). Adherence to Pakistan dietary guidelines–Findings from major cities of Pakistan. medRxiv.

[91] Food and Agriculture Organization of the United Nations (2024). Conflict-Induced Acute Food Crises: Potential Policy Responses to Help Build Resilience.

[92] World Health Organization Regional Office for the Eastern Mediterranean (2020). Health and Well-Being Profile of the Eastern Mediterranean Region: An Overview of the Health Situation in the Region and Its Countries in 2019.

