# Supplementary material for: Food-Based Dietary Guidelines in Countries of the Eastern Mediterranean Region: A Comparison and an Update
Source: Int J Environ Res Public Health. 2025 Nov 26;22(12):1790. doi: 10.3390/ijerph22121790 (PMC13439040; doi:10.3390/ijerph22121790)
Supplement: Supplementary file 1 [file ijerph-22-01790-s001.zip › Proofread Table S1.pdf]

Table S1. The process of FBDG development in countries of the Eastern Mediterranean Region.

Country	Development Process
**Afghanistan [1,2]**	The development of the food-based dietary guidelines (FBDGs) in Afghanistan was a joint, multi-stakeholder process that involved several steps and required diverse skills from interdisciplinary teams representing various government ministries and organizations. It was spearheaded and endorsed by the Ministry of Public Health, the Ministry of Agriculture, Irrigation and Livestock, and the Ministry of Education, in collaboration with the Food and Agriculture Organization (FAO). The process also ensured broad participation from across the population through consultations and focus group discussions, reflecting a participatory and inclusive approach to guideline development.
**Bahrain [3]**	Developed by the Ministry of Health in collaboration with the World Health Organization Regional Office for the Eastern Mediterranean (WHO EMRO).<br>The development of the Bahraini FBDG followed a participatory and evidence-based methodology involving national, international, and technical experts. A triangulated approach was used, consisting of:<br>• An examination of the current situation of the diet-related diseases and food consumption in Bahrain, using published as well as grey literature.<br>• Extensive review and synthesis of cutting-edge and evidence-based research addressing the association of diet with health and diseases, with a focus on the diseases prevalent in Bahrain.<br>• International and local experts' vetting of the science using a culture and context specific lens.<br>A mixed-method approach combining content and thematic analyses was used to synthesize the collected information and to derive the guidelines and recommendations. More specifically, a coding scheme referring to the various themes of dietary recommendations, identified by content analysis, was developed. The thematic analysis was then implemented using this coding scheme to systematically review the various documents, assigning similar pieces of information the same code. The panel of experts examined the results of the coding for synthesis and interpretation, identifying, ranking, and prioritizing the themes that are most relevant within the context of Bahrain.
**Iran [4,5]**	The 2015 version of the Iranian dietary guidelines was prepared by several departments and ministries such as Department of Community Nutrition in the Ministry of Health and Medical Education; with the involvement of the School of Nutritional Sciences and Dietetics, Tehran University of Medical Sciences; Iranian Nutrition Society; and the National Nutrition and Food Technology Research Institute, Tehran. There were also representatives from the other relevant organizations and non-governmental organizations (NGOs). The WHO EMRO supported the process and provided technical assistance.<br><br>The Iranian dietary guidelines are officially endorsed by the Ministry of Health and Medical Education.<br>The development of FBDGs in Iran followed a systematic, participatory process. It began with the collection of data on food consumption patterns, nutrition-related diseases, cultural factors, and food accessibility. Based on these findings, a draft food group

	plan comprising seven groups was prepared and reviewed by experts. After incorporating feedback through meetings and structured interviews, the FBDGs were formulated as clear statements and messages. Pictorial representations were then developed in three designs—pyramid, plate, and rectangle. Finally, the guidelines and visuals underwent preliminary evaluation and field testing among experts, health workers, and community members to assess their acceptability and comprehension.
**Jordan [6]**	The development of the FBDGs was led by the Ministry of Health in Jordan, in collaboration with the WHO EMRO.<br><br>Dietary guidelines were developed by interdisciplinary teams of experts, comprising representatives of health, agriculture, education, nutrition and food science, consumers, NGOs, the food industry, communications and anthropology. Many sources of information were reviewed in this process, such as scientific evidence of the relationship between diet, nutrition and health; and data on food production, food consumption, food composition, cost and accessibility. Once the draft guidelines and food guide were prepared, they were pilot-tested with consumer groups to ensure they are practical and comprehensible and the messages are well-suited to the cultural, social and economic situation of a particular country.
**Kuwait [7]**	The Kuwait FBDGs were developed after consulting experts from renowned institutes in Kuwait, including the Public Authority for Food and Nutrition, Ministry of Health Food and Nutrition Department, Dasman Diabetes Institute, Kuwait Institute for Scientific Research, Kuwait University - College of Public Health and College of Life Sciences. Their collaborative effort and expertise in diverse areas including Public Health Nutrition, Applied Research, Social and Behavioral Science, Clinical Nutrition and Medicine enabled the development of the FBDGs.
**Lebanon [8]**	FBDGs were developed by the Faculty of Agricultural and Food Sciences of the American University of Beirut in collaboration with the Lebanese National Council for Scientific Research (CNRS). The guidelines were adopted by the Ministry of Public Health for its dissemination to policy makers, health-care providers, nutritionists, and dietitians in Lebanon.<br><br>The development of the guidelines was based on sound scientific evidence relating dietary and physical activity practices with health outcomes, and a thorough review of diet-related diseases and conditions in the country. The developed FBDGs have been, therefore, tailored to the food consumption patterns of the Lebanese adult population and the prevalent chronic diseases in the country.
**Oman [9]**	The FBDGs were developed by the Ministry of Health, in collaboration with the WHO EMRO. The guidelines are part of the ministry's strategy to align with the goals of Oman Vision 2040, which prioritizes the health and well-being of citizens and aims to build a society with a high quality of life. The FBDGs were reviewed by a group of experts from the Ministry of Health and other ministries and authorities.
**Pakistan [10]**	Developed by the Ministry of Planning, Development and Reform, with input from academicians, researchers, programme managers, policy makers, development partners and other stakeholders. The development process of the guidelines was in collaboration with FAO and Scaling up Nutrition Movement.<br>Development of dietary guidelines was based on a review of population specific food consumption patterns, dietary habits, nutrients intake, nutrition and health status, socioeconomic conditions and understanding of diet related public health issues of the population.
**Palestine**	--

**Qatar [11]**	The guidelines were developed by the National Dietary Guidelines Task Force composed of public health and nutrition representatives from a wide range of institutions across the country including relevant stakeholders under the direction of the Ministry of Public Health. The guidelines were developed following an evidence-based process, drawing extensively upon key international reports and reviews. Expert committee meetings were held to define food groups, number of servings and serving sizes. Focus groups were conducted to test public understanding of the messages the guidelines were trying to convey. The Qatar Dietary Guidelines are officially endorsed by the Ministry of Public Health.
**Saudi Arabia [12,13]**	Saudi Healthy Plate-2024 was designed using optimization modeling techniques, incorporating global best practices, expert consultations, and rigorous validation processes. The dietary patterns were calibrated to ensure nutritional adequacy and economic feasibility. Monte Carlo simulations with 10,000 iterations were used to evaluate nutrient intake adequacy across various calorie levels (1,600–3,200 kcal). Nutritional constraints and economic assessments adhered to Saudi regulatory standards and data from the General Authority for Statistics.<br>The dietary guidelines were developed by the Ministry of Health (General Directorate of Nutrition). The dietary guidelines development was coordinated by a scientific and technical committee that included an expert group from the Ministry of Health, different ministries, medical institutes and Saudi Food and Nutrition association. This committee was created to work on specific tasks. The Minister of Health endorsed the dietary guidelines.
**UAE [14]**	The Ministry of Health and Prevention developed the Guidelines based on the results of the National Health Survey 2018 and were revised by the National multisectoral Nutrition Committee, which included the Federal Ministry of Health and Prevention, other Emirate-level health authorities, related government sectors, academia, and municipalities. After which, the dietary guidelines were officially endorsed by the Minister of Health and Prevention.
**GCC [15]**	The FBDG were developed through 6 steps: (1) determination of the purpose and goals for establishing FBDG, (2) characteristics of FBDG, (3) determination of the food consumption patterns, (4) review the current nutrition situation, (5) determination of the lifestyle patterns that are associated with diet-related diseases and (6) formulating the FBDG. The FBDG consist of 14 simple and practical pieces of advice taking into consideration the sociocultural status and nutritional problems in the Arab Gulf countries.

Abbreviations: CNRS: National Council for Scientific Research; FAO: Food and Agriculture Organization; FBDGs: food-based dietary guidelines; GCC: Gulf Cooperation Council; NGOs: non-governmental organizations; UAE: United Arab Emirates; WHO EMRO: World Health Organization Regional Office for the Eastern Mediterranean.

References

1. Government of Afghanistan. Afghanistan launches National Food Based Dietary Guidelines (FBDGs) for improved nutrition. Available online: https://reliefweb.int/report/afghanistan/afghanistan-launches-national-food-based-dietary-guidelines-fbdgs-improved (accessed on 10 November 2025).
2. Food and Agriculture Organization; Ministry of Public Health Afghanistan; Ministry of Agriculture Afghanistan; Ministry of Education Afghanistan. National food-based dietary guidelines for Afghans. Available online: https://www.fao.org/3/i5283e/i5283e.pdf (accessed on 20 February 2023).
3. Naja, F.; Khaleel, S.; Alhajeri, M.E.; Ajlan, B.Y.; Abulfateh, N.M.; Alawadhi, A.G.; Bowah, M.H.J.; Al-Jawaldeh, A. The Bahraini food based dietary guidelines: a holistic perspective to health and wellbeing. *Front Public Health* **2023**, *11*, 1182075, doi:doi: 10.3389/fpubh.2023.1182075.
4. Ministry of Health and Medical Education. Food-based dietary guidelines—Iran. Available online: https://www.fao.org/nutrition/education/food-dietary-guidelines/regions/countries/iran/en/ (accessed on 20 February 2023).
5. Safavi, S.; Omidvar, N.; Djazayery, A.; Minaie, M.; Hooshiarrad, A.; Sheikoleslam, R. Development of food-based dietary guidelines for Iran: a preliminary report. *Ann Nutr Metab* **2007**, *51*, 32-35.
6. Al-Jawaldeh, A. Development of food based dietary guidelines. Available online: https://www.researchgate.net/publication/339322960_DEVELOPMENT_OF_FOOD_BASED_DIETARY_GUIDLINES?channel=doi&linkId=5e4b367ba6fdccd965aee258&showFulltext=true (accessed on 10 November 2025).
7. Public Authority for Food and Nutrition. Kuwait food based dietary guideline. Available online: https://www.pafn.gov.kw/assets/images/Uploads/KuwaitFoodbasedDietaryGuidlineEnglish.pdf (accessed on 20 February 2023).
8. The American University of Beirut / Lebanese National Council for Scientific Research / Ministry of Public Health. The Food-Based Dietary Guideline Manual for Promoting Healthy Eating in the Lebanese Adult Population. Available online: https://www.fao.org/nutrition/education/food-dietary-guidelines/regions/countries/lebanon/en/ (accessed on 20 February 2023).
9. Ministry of Health-Sultanate of Oman. The Omani Guide for Healthy Eating. Available online: https://moh.gov.om/media/c3vhtd2y/oman-diatry-guideline-english-feb-19.pdf (accessed on 12 August 2025).
10. Food and Agriculture Organization of the United Nations; Ministry of Planning/Development and Reform/Government of Pakistan. Pakistan Dietary Guidelines for Better Nutrition. Available online: https://www.pc.gov.pk/uploads/report/Pakistan_Dietary_Nutrition_2019.pdf (accessed on 9 October 2022).
11. The Supreme Council of Health. Qatar Dietary Guidelines. Available online: https://www.fao.org/3/az908e/az908e.pdf (accessed on 22 February 2023).
12. Alhumaidan, O.A.; Alkhunein, S.M.; Alakeel, S.A.; Fallata, G.A.; Aldhwayan, M.M.; Alfaifi, A.Y.; Albalwi, W.M.; AlZeer, H. Saudi healthy plate-2024: framework for developing, modeling, and evaluating Saudi Arabia's dietary guidelines. *BMC Nutr* **2025**, *11*, 160.

13. Ministry of Health Kingdom of Saudi Arabia. Dietary guidelines for Saudis: The healthy food palm. Available online: https://www.fao.org/nutrition/education/food-dietary-guidelines/regions/countries/saudi-arabia/en/ (accessed on 21 February 2023).
14. United Arab Emirates Ministry of Health and Prevention. United Arab Emirates Dietary Guidelines (Arabic). Available online: https://www.fao.org/nutrition/education/food-dietary-guidelines/regions/countries/united-arab-emirates/en/ (accessed on 22 February 2023).
15. Musaiger, A.O.; Takruri, H.R.; Hassan, A.S.; Abu-Tarboush, H. Food-based dietary guidelines for the Arab Gulf countries. *J Nutr Metab* **2012**, *2012*, 905303.
