# Supplementary material for: Food-Based Dietary Guidelines in Countries of the Eastern Mediterranean Region: A Comparison and an Update
Source: Int J Environ Res Public Health. 2025 Nov 26;22(12):1790. doi: 10.3390/ijerph22121790 (PMC13439040; doi:10.3390/ijerph22121790)
Supplement: Supplementary file 1 [file ijerph-22-01790-s001.zip › Proofread Table S2.pdf]

Table S2. Examples of culture-specific foods in the FBDGs.

Country	Examples of Culture-Specific Foods
**Afghanistan [1]**	Afghani dishes such as rice, curd and mingbeans together (kechery krut), pumpkin and chickpeas (qorma kadu), eggplant with yogurt (banjan krut)<br>Deep-fried chips like bullani<br>Milk products like doogh, chaka and qorut<br>Naan bread<br>Nabat (sugar lumps or rock candy)<br>Pickling vegetables to make atchars<br>Vegetables like bulan, shurak and sech<br>Other examples include halwa and ferni
**Bahrain [2]**	Dairy products like laban, labneh, halloumi and baladi cheeses<br>Fatty fish such as maid, chan'ad, yanam<br>High fat dish such as Baja<br>Mahyawa<br>Markouk bread<br>Sheera and dibs<br>Traditional dishes such as Majbous, Salona, Biryani, Harees and Madhrooba<br>Traditional legume-based dishes such as hummus<br>Traditional sweets such as Balaleet, Khanfaroush, Rankayna and Darmanda, Aseeda and Sago
**Iran**	--
**Jordan [3]**	Dairy products such as laban and kashta<br>Freekeh<br>Hammam bread<br>Shawarma<br>Traditional beverages such as Amardeen drink<br>Traditional sweets such as Baklava and Knefeh<br>Zaatar

**Kuwait [4]**	Dairy products such as laban, labneh, halloumi and akkawi cheeses<br>Maed fish<br>Tannour bread<br>Traditional dishes like harees and jareesh<br>Traditional legume-based dishes such as fava beans, chickpeas, hummus, as well as falafel<br>Traditional sweets such as Luqaimat<br>Zaatar
**Lebanon [5]**	Arabic pita bread<br>Arabic sweets such as Ma'amool and Baklava and Katayef<br>Dairy products such as labneh, halloumi, akkawi, double crème and mjaddal cheeses<br>Kaak and Ka'ak Ourshalli<br>Lebanese bread<br>Milk-based puddings such as Mhalbiyeh, Sahlab or Riz Bi Halib<br>Traditional beverages such as Jellab and Tamir Hindi<br>Traditional dip-foods such as Hummus bi Tahini<br>Traditional dishes such Mjaddara, Mdardara, Riz bi Hummus or Riz bi Fool, Fattet Hummus, Baleela, Fool Mdammas, Shish Barak and Kibbeh bi Laban<br>Traditional hot dishes such as vegetables stuffed with rice and meat, and vegetable-based stews that are usually consumed with rice<br>Traditional salads such as Fattoush, Tabbouleh, fresh Zaatar salad<br>Other examples include Kamhyeh, Sawda, Nkhaa, Shawarma, Makanik, Sujuk and Falafel
**Oman [6]**	Arabic sweets such Goallah wa Asal, Khabisah, Aseedah, Luquimat, Qashati, Maho, Basboosah, Knefeh, Baklawa, Maa'mool<br>Dairy products such as laban, labneh, laban ayran, kashta, halloumi, akkawi, baladi and kami cheeses<br>Dates (Majdoul, Barhi, khalas, barni or jabri dates)<br>Dhofari kaak<br>Harees wheat, greesh, shorbet hab<br>Hummus<br>Manaeesh<br>Milk-based puddings such as Mhalbiyeh

	Omani bread<br>Traditional beverages such as Tamir Hindi, Jellab, Karkadeh<br>Traditional dishes such as Qabooli, Biryani, Mandi, Madhrooba, Kabsa<br>Traditional fish such as Awal and Qashaa and Maleh<br>Traditional salads such as Fattoush, Tabbouleh, fresh Zaatar salad<br>Wheat-based desserts such as Sago, Sakhanet al Habba al Hamra and Sakhanet al Loubia<br>Other examples include<br>• Falafel, sambousik<br>• Mjaddara, Mdardara, Fasoulia, Nashef Chickpeas, Nashef Beans, Fattet Hummus, Chickpeas and Fool Mdammas<br>• Mashakek, Mathbi, Makli Laham, Mahamas, Shuwa, Mashawi Laham, Saloona Laham, Kabsa Laham, Shawarma, Kebbeh<br>• Catashory, Catalase, Mandazy, Lowlah
**Pakistan [7]**	Dairy products such as kheer or feerni or other milk-based products<br>Dal, shami kabab<br>Kitchri<br>Naan bread<br>Pakistani chapatti<br>Pakoras, chat, cholay, samosas<br>Pulao, Raita<br>Suji<br>Traditional beverages such as Lassi drink
**Palestine [8]**	Arabic bread<br>Dairy products such as laban, labneh and kashta<br>Freekeh<br>Hammam bread<br>Hummus<br>Milk-based puddings such as Mhalbiyeh, Sahlab or Riz Bi Halib<br>Nigella seeds (habbel el barake)<br>Stuffed meals such as kebbe<br>Traditional beverages such as Tamir Hindi

	Zaatar
**Qatar [9]**	Dairy products such as laban, labneh, laban ayran, halloumi and akkawi cheeses<br>Fish such as zobaidy (malabar cavalla) and gubgub<br>Fried breads (paratha, puri)<br>Hummus<br>Milk-based desserts such as Mhalbiyeh and Umm Ali<br>Traditional beverages such as Karak drink<br>Traditional dishes such as foul mudammes, hummus, bajela and nakhi<br>Traditional salads such as Fattoush and Tabbouleh<br>Other examples include Jareesh, Makdous, Fatayer, Falafel, Nakanik and Sujuk
**Saudi Arabia [10]**	Arabic bread<br>Dairy products such as laban<br>Dates
**UAE [11]**	Dairy products such as robb<br>Falafel<br>Irani bread<br>Lebanese bread<br>Milk-based puddings such as Mhalbiyeh<br>Sujuk<br>Zaatar

Abbreviations: FBDGs: food-based dietary guidelines; UAE: United Arab Emirates.

References

1. Food and Agriculture Organization; Ministry of Public Health Afghanistan; Ministry of Agriculture Afghanistan; Ministry of Education Afghanistan. National food-based dietary guidelines for Afghans. Available online: https://www.fao.org/3/i5283e/i5283e.pdf (accessed on 20 February 2023).
2. Ministry of Health-Bahrain. Food Based Dietary Guidelines for the Kingdom of Bahrain. Available online: https://www.moh.gov.bh/Content/Upload/File/638422157715028884-225MOH---The-Food-National-Based-Dietary-Guidelines_.pdf (accessed on 12 August 2025).
3. Ministry of Health (MOH) Jordan. Food Based Dietary Guideline for Jordanians (Arabic). Available online: https://www.moh.gov.jo/ebv4.0/root_storage/ar/eb_list_page/%D8%A7%D9%84%D8%AF%D9%84%D9%8A%D9%84_%D8%A7%D9%84%D8%BA%D8%B0%D8%A7%D8%A6%D9%8A_%D9%84%D9%84%D8%A7%D8%B1%D8%AF%D9%86%D9%8A%D9%86.pdf (accessed on 12 August 2025).
4. Public Authority for Food and Nutrition. Kuwait food based dietary guideline. Available online: https://www.pafn.gov.kw/assets/images/Uploads/KuwaitFoodbasedDietaryGuidlineEnglish.pdf (accessed on 20 February 2023).
5. The American University of Beirut / Lebanese National Council for Scientific Research / Ministry of Public Health. The Food-Based Dietary Guideline Manual for Promoting Healthy Eating in the Lebanese Adult Population. Available online: https://www.fao.org/nutrition/education/food-dietary-guidelines/regions/countries/lebanon/en/ (accessed on 20 February 2023).
6. Ministry of Health-Sultanate of Oman. The Omani Guide for Healthy Eating. Available online: https://moh.gov.om/media/c3vhtd2y/oman-diatry-guideline-english-feb-19.pdf (accessed on 12 August 2025).
7. Food and Agriculture Organization of the United Nations; Ministry of Planning/Development and Reform/Government of Pakistan. Pakistan Dietary Guidelines for Better Nutrition. Available online: https://www.pc.gov.pk/uploads/report/Pakistan_Dietary_Nutrition_2019.pdf (accessed on 9 October 2022).
8. Ministry of Public Health Palestine; World Health Organization. *Palestinian food based dietary guidelines (Arabic)*; 2021.
9. The Supreme Council of Health. Qatar Dietary Guidelines. Available online: https://www.fao.org/3/az908e/az908e.pdf (accessed on 22 February 2023).
10. Ministry of Health Kingdom of Saudi Arabia. Dietary guidelines for Saudis: The healthy food palm. Available online: https://www.fao.org/nutrition/education/food-dietary-guidelines/regions/countries/saudi-arabia/en/ (accessed on 21 February 2023).
11. United Arab Emirates Ministry of Health and Prevention. United Arab Emirates Dietary Guidelines (Arabic). Available online: https://www.fao.org/nutrition/education/food-dietary-guidelines/regions/countries/united-arab-emirates/en/ (accessed on 22 February 2023).
